# Transcriptome Analysis Reveals Changes in Whole Gene Expression, Biological Process, and Molecular Functions Induced by Nickel in Jack Pine (*Pinus banksiana*)

**DOI:** 10.3390/plants12152889

**Published:** 2023-08-07

**Authors:** Alistar Moy, Karolina Czajka, Paul Michael, Kabwe Nkongolo

**Affiliations:** Biomolecular Sciences Program and Department of Biology, School of Natural Sciences, Laurentian University, Sudbury, ON P3E 2C6, Canada; amoy@laurentian.ca (A.M.); kczajka@laurentian.ca (K.C.); pmichael@laurentian.ca (P.M.)

**Keywords:** *Pinus banksiana*, nickel stress, whole genome expression, biological process, molecular function, cellular compartment

## Abstract

Understanding the genetic response of plants to nickel stress is a necessary step to improving the utility of plants in environmental remediation and restoration. The main objective of this study was to generate whole genome expression profiles of *P. banksiana* exposed to nickel ion toxicity compared to reference genotypes. *Pinus banksiana* seedlings were screened in a growth chamber setting using a high concentration of 1600 mg of nickel per 1 kg of soil. RNA was extracted and sequenced using the Illumina platform, followed by de novo transcriptome assembly. Overall, 25,552 transcripts were assigned gene ontology. The biological processes in water-treated samples were analyzed, and 55% of transcripts were distributed among five categories: DNA metabolic process (19.3%), response to stress (13.3%), response to chemical stimuli (8.7%), signal transduction (7.7%) and response to biotic stimulus (6.0%). For molecular function, the highest percentages of genes were involved in nucleotide binding (27.6%), nuclease activity (27.3%) and kinase activity (10.3%). Sixty-two percent of genes were associated with cellular compartments. Of these genes, 21.7% were found in the plasma membrane, 16.1% in the cytosol, 12.4% with the chloroplast and 11.9% in the extracellular region. Nickel ions induced changes in gene expression, resulting in the emergence of differentially regulated categories. Overall, there were significant changes in gene expression with a total 4128 genes upregulated and 3754 downregulated genes detected in nickel-treated genotypes compared to water-treated control plants. For biological processes, the highest percentage of upregulated genes in plants exposed to nickel were associated with the response to stress (15%), the response to chemicals (11,1%), carbohydrate metabolic processes (7.4%) and catabolic processes (7.4%). The largest proportions of downregulated genes were associated with the biosynthetic process (21%), carbohydrate metabolic process (14.3%), response to biotic stimulus (10.7%) and response to stress (10.7%). For molecular function, genes encoding for enzyme regulatory and hydrolase activities represented the highest proportion (61%) of upregulated gene. The majority of downregulated genes were involved in the biosynthetic processes. Overall, 58% of upregulated genes were located in the extracellular region and the nucleus, while 42% of downregulated genes were localized to the plasma membrane and 33% to the extracellular region. This study represents the first report of a transcriptome from a conifer species treated with nickel.

## 1. Introduction

Nickel mining and processing have occurred in the Greater Sudbury region of Ontario for more than 130 years [1,2,3]. Despite the large environmental risks, mining activity is expected to increase nickel production to keep up with rising global demand. Nickel contamination causes considerable damage to plant biota, animal communities and ecosystems [4,5]. In plants, nickel is an essential micronutrient at low levels [6]. At higher concentrations, nickel has been found to diminish photosynthesis by decreasing the functionality of photosystem II and by inhibiting chlorophyll function and production [7,8,9,10]. Excess nickel causes severe dysfunction of homeostasis for many metals, including copper, iron, manganese and zinc, resulting in a variety of physiological problems corresponding to those metals [11,12,13]. Unlike other metals, nickel indirectly causes the overproduction of reactive oxygen species (ROS) by increasing or decreasing the activity of antioxidative enzymes, such as superoxide dismutase (SOD) and catalase (CAT) [5,14]. The inhibition of these detoxifying enzymes hinders the overall growth and development of the plant [10,15,16]. Jack pine (*Pinus banksiana*) has been proposed as a candidate species for regreening and remediation efforts due to its acclimation to the cold and growth in challenging climates. Additionally, Jack pine has been successfully used in a regreening project in the Sudbury region [17].

Mechanisms involved in nickel resistance and detoxification remain poorly elucidated in comparison to other heavy metals, such as copper. In response to excess heavy metals, plants may modulate the production of chelators, metallothionein and transporter proteins in their tissues. Plants may also regulate antioxidative enzymes in response to ROS produced as by-products of heavy metal toxicity. In response to excess nickel, genes encoding the chelators nicotianamine and histidine were found to be upregulated in the hyperaccumulators *Thlaspi caeulescens* and *Alyssum lesbiacum*, respectively [18,19]. The IREG2 transporter gene has been found to be upregulated in the hyperaccumulators *Psychotria gabriellae* and *Noccaea japonica*, suggesting nickel sequestration into the vacuoles of root cells during the initial uptake of nickel into the roots [20,21]. In *Pinus banksiana* and *Pinus strobus*, excess nickel prompted downregulation of the gene encoding the natural resistance-associated macrophage protein (NRAMP3) [22]. In contrast, this gene was upregulated in *Picea glauca* under conditions of excess nickel [23]. In some species, NRAMP3 is localized to the vacuole membrane, implying a possible role in nickel sequestration into the vacuole [24,25]. Although the expression of particular genes, such as NRAMP3, has been studied in conifers, the extent to which the genes are expressed or regulated relative to other genes remains elusive. This study is the first to characterize and describe the transcriptome in a nickel-treated coniferous tree, providing indispensable information to other researchers for understanding conifer genetics and responses to nickel stress.

The objectives of this study were to: (1) characterize the transcriptome of Jack pine (*Pinus banksiana*); and (2) use transcriptome analysis and gene ontology to characterize gene expression in response to nickel stress.

## 2. Materials and Methods

### 2.1. Plant Treatment

*Pinus banksiana* seedlings were provided by the College Boreal Plant Center located in Sudbury, Ontario. The six-month-old seedlings were transplanted into pots containing a 1:1 mixture of sand and a mixture of 79% Sphagnum moss, 17% perlite and 5% composted peat moss and grown in a growth chamber for one month (Figure S1). The plants were fertilized with a 1:1:1 mixture of nitrogen, phosphorous and potassium fertilizer when required. After one month, the seedlings were treated and then placed back in the growth chamber in a randomized block design. Fifteen seedlings were treated with 1600 mg of nickel sulfate per 1 kg of soil. This treatment represented the infield concentration of Ni from a survey on metal-contaminated sites in the Greater Sudbury Region [26]. Ten seedlings were given deionized water, representing the negative controls. Five seedlings were treated with an equimolar concentration of potassium sulfate corresponding to the sulfate ion portion of the 1600 mg/kg concentration of nickel sulphate. The seedlings were grown for an additional two weeks post-treatment. Needles from the selected seedlings were harvested. For longer-term storage, the needles were flash frozen using liquid nitrogen and stored in a freezer at −80 °C.

### 2.2. RNA Extraction

RNA extraction was performed on the needles of the seedlings following the protocol from the NORGEN BIOTEK Plant/fungi total RNA purification kit, which can be found here: https://norgenbiotek.com/product/plantfungi-total-rna-purification-kit, (accessed on 1 January 2022). Agarose gel electrophoresis was performed on the extracted RNA to assess RNA quality. The quantity of RNA for each sample was determined using the Qubit™ RNA BR assay kit. The extracted RNA samples were stored in a freezer at −80 °C.

### 2.3. RNA Sequencing and De Novo Transcriptome Assembly

Messenger RNA (mRNA) was isolated from total RNA. RNA chemical fragmentation was performed to account for the size limitations of the sequencing platform. Then, mRNA was reverse transcribed to cDNA using reverse transcriptase, and RNAse was added to prevent unnecessary ligation between different nucleotide strands. Second strand synthesis was performed, followed by 3′ end ligation with adaptors and adenosine caps. The cDNA was amplified to generate cDNA libraries. Illumina sequencing (performed at Seqmatic in San Francisco, CA, USA) was used to sequence the cDNA libraries. FASTQC files containing the raw reads of each cDNA library were generated for each sample. The FASTQC program verified the quality of raw data from the files and provided attributes for each sequence, which included average sequence length, %GC content, total deduplicated percentage and sequences flagged as poor quality. The Cutadapt program was used to remove adaptor sequences and low-quality bases from the raw read data. The Bowtie2 algorithm in Trinity was used to map RNA sequence raw reads to the Trinity transcript assembly, generating sequence alignment map (SAM) files that were then converted into BAM (binary form of SAM) files. Transcript assembly was performed by inputting RNA sequence data from all samples into the Trinity program, which quantified the number of genes based on the number of detected isoforms.

### 2.4. BLAT Matching and Annotation of Pinus banksiana Genes

Transcripts were characterized by performing two-way BLAST-like alignment tool (BLAT) matching with the *Pinus taeda* genome as a reference. Attributes such as transcript ID, gene ID and corresponding log (E-value) for sequence similarity with the reference genome were characterized. Other identified characteristics identified by BLAT matching included query sequence size, transcript sequence size and the percentage of net match for each characteristic. Every transcript was mapped to protein sequences in the UniProt database, generating corresponding UniProt IDs. Protein matches with the highest degree of similarity were used to annotate genes and assign gene ontology information, such as gene description.

### 2.5. Quantification of Gene Expression and Quality Control (QC) Analysis

The RNA-Seq by Expectation-Maximization (RSEM) abundance estimation method was used to quantify the expression level of each gene/transcript and related isoforms. Quality control for read counts was performed to critically assess the number of counts from each gene. Raw reads were filtered and selected for counts of at least 1, 2, 10, 50 or 100. Genes with 1 read were considered noise. Genes with 2 or more counts were used as an estimate of the number of genes expressed. Genes with 10 or more counts were considered an adequate indication of the number of genes that had enough reads for downstream statistical analysis. For each treatment group, genes with a counts per million (CPM) value of 1 or higher in at least two samples were included in downstream analysis. Genes with a CPM value of less than 1 in at least two samples were unexpressed and removed. Normalization factors for raw counts were generated using a trimmed mean of M-values (TMM) from edge R to remove variations from samples and normalize the samples.

The normalized read counts were log-scale transformed using the voom method (log2 scale) from the limma package in R. Boxplots of the transformed expression values were generated to show the mean distribution of every sample. Deviation from the mean distribution in a particular sample may indicate variations among experimental conditions, sample contamination or batch effects. Samples that deviated significantly from the mean distribution within the same objective group were excluded.

Multidimensional scaling plots were generated to display the clustering of sample groups based on the leading logFC of the normalized data. Groups of samples that deviated significantly from other groups of samples were considered differentially regulated. Samples that deviated significantly from the other samples within the same group were considered outliers and were not included in downstream analysis.

**Figure 1:**
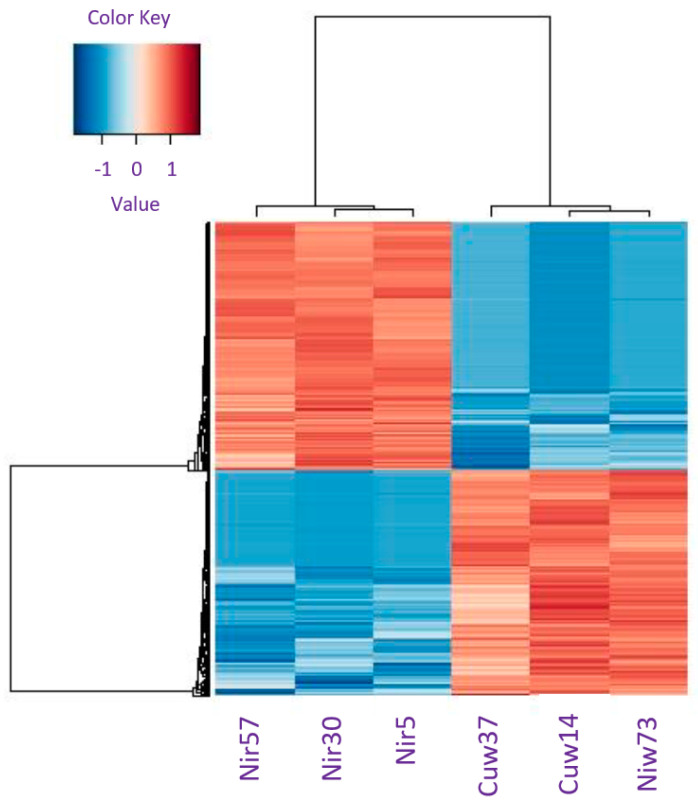
Heatmap of differentially expressed genes from nickel-treated plants compared to controls in *Pinus banksiana*. Differentially expressed gene values are based on the Log2 normalized FC, with red cells representing upregulation and blue cells representing downregulation. Nickel-treated individuals are labeled Nir57, Nir30 and Nir5. Water controls are labeled Cuw37, Cuw14 and Niw73.

A heatmap was generated from the logFC of 5000 genes to show the relationship of gene expression between the samples. Samples that did not have a similar logFC were considered outliers and were not included in the downstream analysis. The proportion of raw reads expressed by the top 100 upregulated and downregulated genes were also assessed in every sample to identify potential bottlenecking issues (Figure 1).

### 2.6. Differential Gene Expression (DGE) Analysis of Pairwise Comparisons

The cutoff for pairwise comparisons was calculated to be equivalent to 10 raw counts. From the average of total counts in all samples, a CPM of 0.361 was calculated as the minimum threshold required to be included in pairwise comparisons. Genes that had a CPM higher than the cutoff in at least two samples were included in the downstream analysis, whereas genes that did not fulfill these parameters were excluded. The pairwise comparisons of transcripts were performed between treated samples and the controls. Differential gene expression, expressed as logFC values, was evaluated using the limma package in R. To assess the interference of sulfate ions on the treatment regimen, pairwise comparisons of expressed genes were also conducted between nickel-treated plants and the potassium controls and between the water and potassium controls. The entire set of genes for each pairwise comparison was annotated using Trinotate and Trinity. Gene ontology was performed by assigning GO terms and gene IDs from available databases to the set of genes for a particular pairwise comparison. Genes that could not be annotated were filtered out of the set of annotated genes. Each gene set was run through a plant slim function using the Omicsbox program. Gene ontology charts functionally categorizing biological processes, metabolic functions and cellular components were generated. For each functional category, sequences were distributed using the NodeScore of each assigned GO term.

### 2.7. Analysis of Top Differentially Regulated Genes

The top 100 upregulated genes and downregulated genes were ranked between the nickel-treated plants and controls. Genes were ranked based on LogFC and fulfillment of high stringency parameters. UniProt annotation and a review of the current literature were performed to characterize genes associated nickel detoxification tolerance mechanisms. Genes associated with nickel resistance were considered candidate genes. Gene ontology charts functionally categorizing biological processes, metabolic functions and cellular component localization were generated for the top 100 regulated genes using the aforementioned process in DGE analysis. Charts comprising the top 25 genes were provided for each pairwise comparison.

The top 100 genes for each pairwise comparison were obtained from the set of differentially expressed genes and categorized into upregulated and downregulated values. Genes with the highest or lowest expression were correlated with nickel stress and could be used to partially describe the genetic response to nickel. Protein descriptions with the “predicted protein” label indicated no assignment of any closely related protein or relevant GO terms from the UniProt database. Gene ontology terms and functional categorizations were assigned by the Omics Box/BLAST2GO program.

## 3. Results

### 3.1. Transcript Assembly and Sequence Data QC

The FastQC program characterized the raw reads from Illumina sequencing and verified the quality of the data. None of the sequences were flagged as poor quality. Nickel-treated plants had 35–51 million total sequences. The average sequence length was 51 bases. Nickel-treated samples had a total deduplicated percentage of 24–41%, indicating that a significant proportion of the gene expression was from duplicated gene expression.

### 3.2. Differential Gene Expression (DGE) Analysis

This transcriptome shotgun assembly project has been deposited in the NCBI BioProject database under accession number PRJNA962116. Overall, 581,037 transcripts were mapped to protein sequences in the UniProt database, and the closest matches were used to annotate genes. Overall, 25,552 transcripts were assigned gene ontology. A multidimensional scale plot and hierarchical cluster map were used to assess the clustering between samples. The water and potassium controls clustered close to each other, indicating that gene expression was similar between the treatment groups and that sulfate had a negligible effect on the treatment regimen. Clustering between individuals did not indicate the presence of potential outliers. Expression of nickel-treated samples was significantly different from the water and potassium controls (Table 1). DEGs only from the high stringency cutoff (two-fold and FDR 0.05) were considered. Hierarchical clustering in all samples indicated that the samples within each treatment group were more similar to each other than to samples from other treatment groups.

### 3.3. Gene Ontology Classification of Differentially Expressed Genes in Pinus banksiana

**Figure 2:**
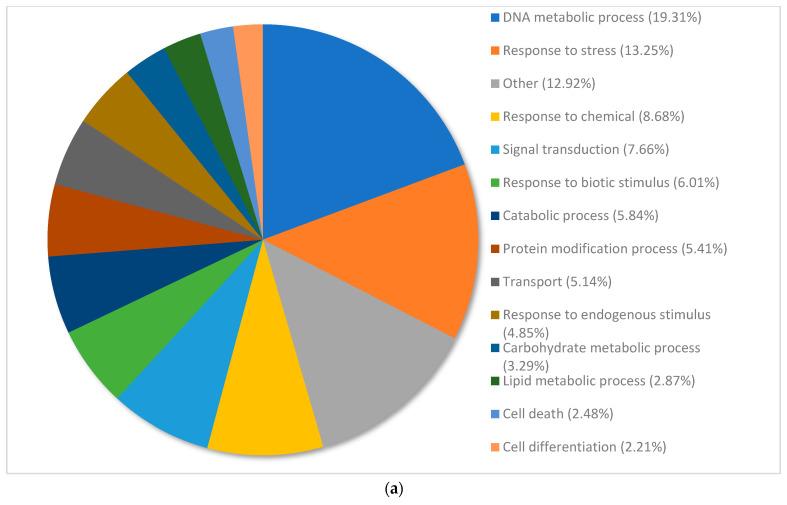

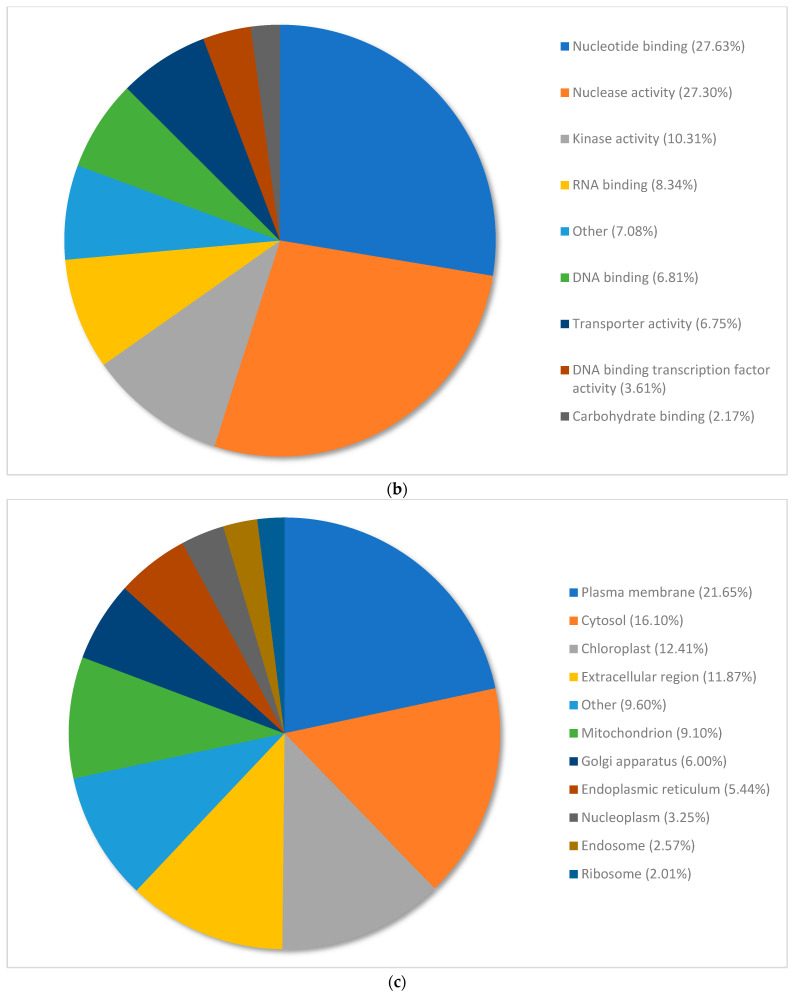
(**a**) Percentage of annotated transcripts in *Pinus banksiana* control samples categorized by biological processes. A total of 5112 transcripts from the water controls were grouped by gene ontology terms within the biological processes category using Omicsbox (BLAST2GO). Terms with less than 2% of total gene expression were combined and assigned the label “other”. (**b**) Percentage of annotated transcripts in *Pinus banksiana* control samples categorized by molecular function. A total of 3755 transcripts from the water controls grouped by gene ontology terms within the molecular function category using Omicsbox (BLAST2GO). Terms with less than 2% of total gene expression were combined and assigned the label “other”. (**c**) Percentage of annotated transcripts in *Pinus banksiana* control samples categorized by cellular component. A total of 3385 transcripts from the water controls grouped by gene ontology terms within the cellular component using Omicsbox (BLAST2GO). Terms with less than 2% of total gene expression were combined and assigned the label “other”.

Gene ontology graphs show the distributions of annotated genes to different terms within the following categories: biological processes, metabolic function and cellular compartment (Figure 2a–c). The proportion of genes allocated to each term was similar among the water controls and treated plants.

Overall, 5112 transcripts were annotated and categorized in biological processes. Detailed transcriptome analysis showed that 54.91% of transcripts were categorized under the following terms: DNA metabolic process (19.31%), response to stress (13.25%), response to chemicals (8.68%), signal transduction (7.66%) and response to biotic stimulus (6.01). Response to stress, response to chemicals, and response to biotic stimulus were among the top 5 terms with the most expression that fell under the parent category of response to stimulus. Eighteen (18) terms had less than 2% of the distribution of genes and were collectively assigned to the category “other” (Figure 2a).

Overall, 3755 transcripts were annotated and categorized by molecular function. Of these transcripts, 65.24% were allocated to the following terms: nucleotide binding (27.63%), nuclease activity (27.30%) and kinase activity (10.31). Five of eight categories were related to nucleotide function and genetic regulation. Nucleotide binding, nuclease activity, RNA binding and DNA binding represented four of the top five categories, indicating the prominence of nucleotide function and genetic regulation in top regulated genes. Additionally, nucleotide binding, RNA binding and DNA binding fell under the parent category of nucleic acid binding. Nine terms had less than 2% of total gene expression and were collectively assigned to the category “other” (Figure 2b).

Overall, 3385 transcripts were annotated and categorized based on cellular compartment location. Of these transcripts, 62.03% of genes were categorized under the following terms: plasma membrane (21.65%), cytosol (16.10%), chloroplast (12.41%) and extracellular region (11.87%). Plasma membrane, cytosol and the extracellular region represented three of the top five categories, which were relegated to compartments encompassing or adjacent to the plasma membrane. Seven categories had less than 2% of the distribution of genes and were collectively assigned to the category “other (Figure 2c).

### 3.4. Gene Ontology of the Top 100 Differentially Expressed Genes in Pinus banksiana

**Figure 3:**
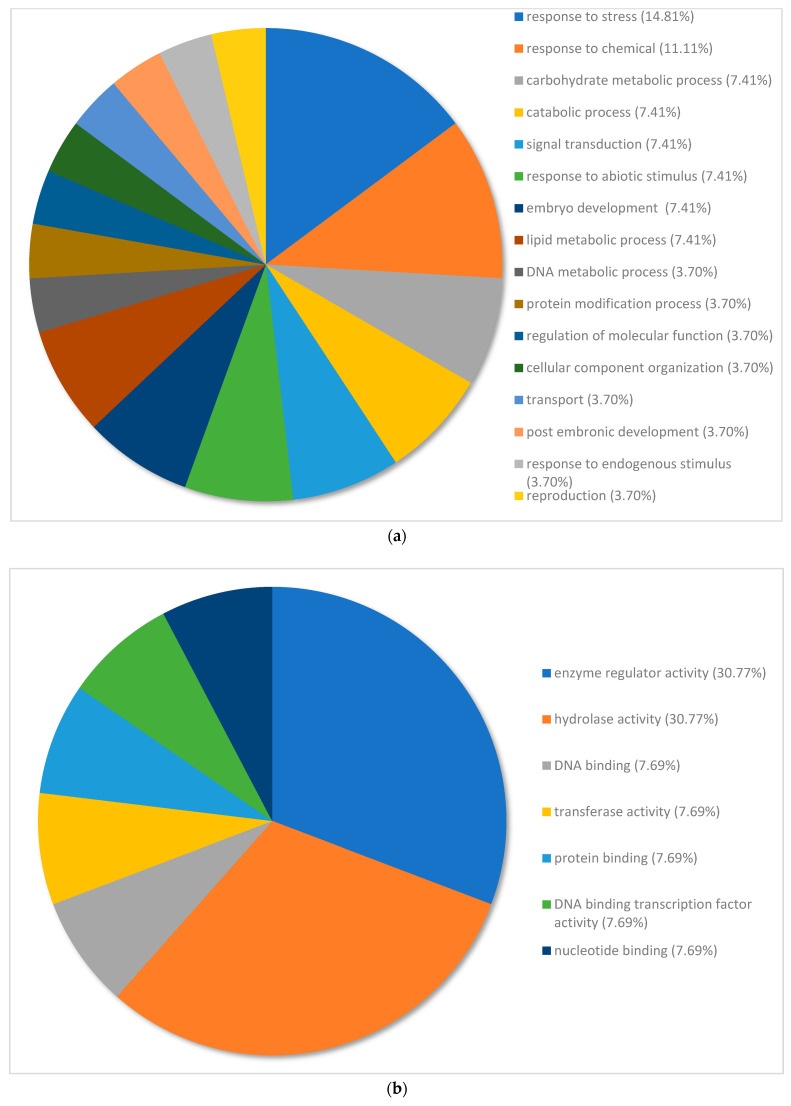

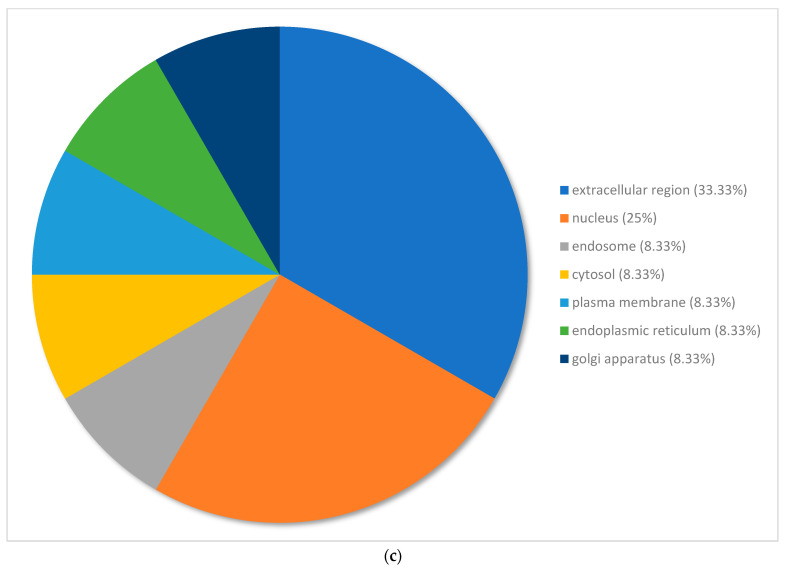
(**a**) Percentage of the top 100 upregulated transcripts in *Pinus banksiana* nickel-treated plants compared to the controls categorized by biological processes. A total of 100 transcripts from nickel-treated samples compared to water controls were grouped by gene ontology terms within the biological processes category using Omicsbox (BLAST2GO). Terms with less than 2% of total gene expression were combined and assigned the label “other”. (**b**) Percentage of the top 100 upregulated transcripts in *Pinus banksiana* nickel-treated plants compared to the controls categorized by molecular function. A total of 100 transcripts from nickel-treated samples compared to the water controls were grouped by gene ontology terms within the molecular function category using Omicsbox (BLAST2GO). Terms with less than 2% of total gene expression were combined and assigned the label “other”. (**c**) Percentage of the top 100 upregulated transcripts in *Pinus banksiana* nickel-treated plants compared to the controls categorized by cellular component. A total of 100 transcripts from the treated samples compared to water controls were grouped by gene ontology terms within the cellular component category using Omicsbox (BLAST2GO). Terms with less than 2% of total gene expression were combined and assigned the label “other”.

**Figure 4:**
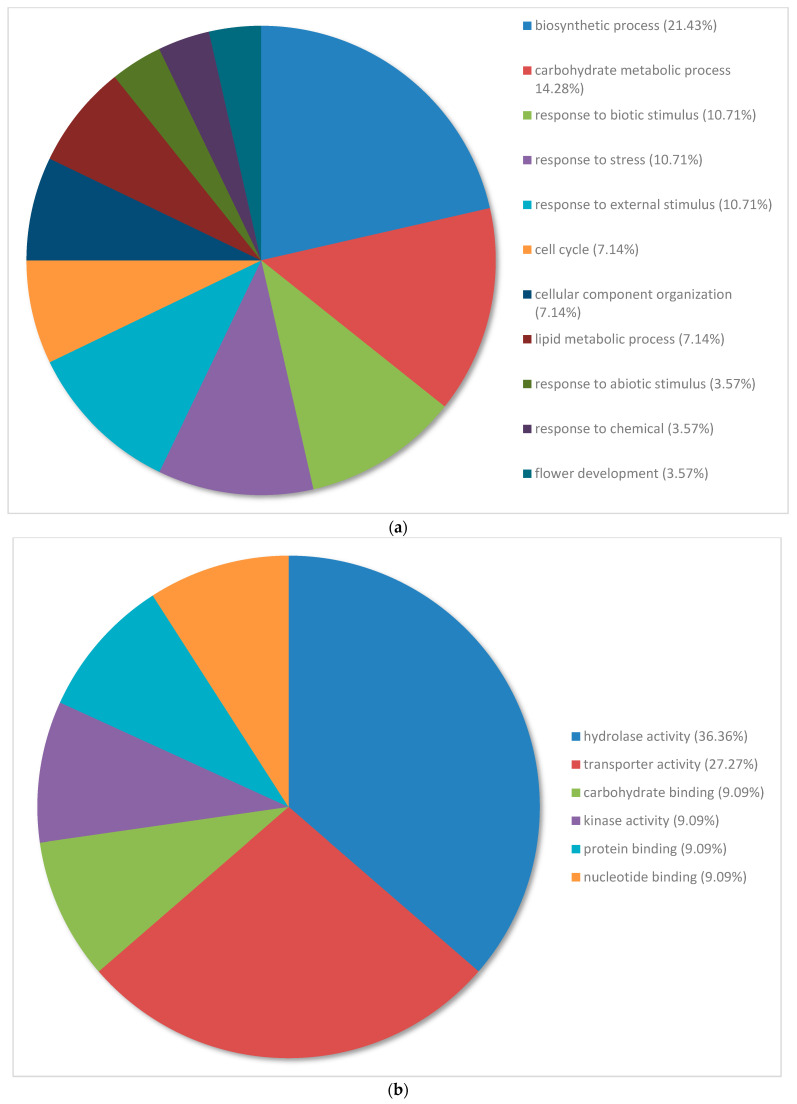

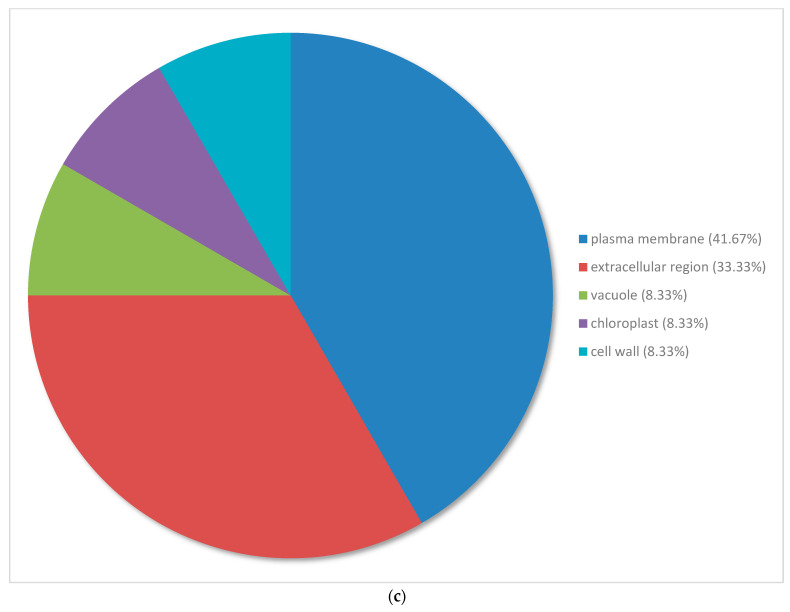
(**a**) Percentage of the top 100 downregulated transcripts in *Pinus banksiana* nickel-treated plants compared to water controls categorized by biological processes. A total of 100 transcripts from the treated samples compared to the water controls were grouped by gene ontology terms within the biological processes category using Omicsbox (BLAST2GO). Terms with less than 2% of total gene expression were combined and assigned the label “other”. (**b**) Percentage of the top 100 downregulated transcripts in *Pinus banksiana* nickel-treated plants compared to water controls categorized by molecular function. A total of 100 transcripts from the treated samples compared to the water controls were grouped by gene ontology within the molecular function category using Omicsbox (BLAST2GO). Terms with less than 2% of total gene expression were combined and assigned the label “other”. (**c**) Percentage of the top 100 downregulated transcripts in *Pinus banksiana* nickel-treated plants compared to water controls categorized by cellular component. A total of 100 transcripts from treated samples compared to the water controls were grouped by gene ontology terms in the cellular component category using Omicsbox (BLAST2GO). Terms with less than 2% of total gene expression were combined and assigned the label “other”.

Gene ontology graphs show the distribution of the top 100 genes allocated to different terms within the categories of biological processes, metabolic function and cellular compartment (Figure 3 and Figure 4).

The 100 most upregulated genes in the nickel-treated group compared to the water control group were annotated and categorized based on biological processes (Figure 3a). Overall, 70.38% of genes were distributed under the following processes: response to stress (14.81%), response to chemicals (11.11%), carbohydrate metabolic process (7.41%), catabolic process (7.41%), signal transduction (7.41%), response to abiotic stimulus (7.41%), embryo development (7.41%) and lipid metabolic process (7.41%). Response to stress, response to chemicals and response to abiotic stimulus fell under the same parent category of response to stimulus. Compared to the entire transcriptome, DNA metabolic process had a smaller percentage of expressed genes. In contrast, carbohydrate metabolic process and lipid metabolic process had large proportions of expressed genes but were underrepresented in the entire transcriptome. Embryo development, postembryonic development and reproduction were also represented in this instance but had less than 2% of expressed genes in the entire transcriptome.

The 100 most upregulated genes in the nickel-treated group compared to the water control group were categorized based on molecular function. Overall, 61.54% of genes were categorized under the following molecular functions: enzyme regulator activity (30.77%) and hydrolase activity (30.77%). Enzyme regulatory activity and hydrolase activity comprised the majority of top upregulated genes despite having less than 2% of genes in the entire transcriptome. Protein binding and transferase activity were also represented among the top upregulated genes despite comprising less than 2% of the whole transcriptome (Figure 3b).

The 100 most upregulated genes in nickel-treated samples compared to water controls were annotated and categorized based on cellular compartment. Overall, 58.33% of genes were categorized under the following cell compartments: extracellular region (33.33%) and nucleus (25%). Other organelles had an equal distribution of expressed genes. In contrast to the whole transcriptome, the nucleus comprised a very large portion of expressed genes (Figure 3c).

The 100 most downregulated genes in the nickel-treated group compared to the water control group were annotated and categorized based on biological process (Figure 4a). They were categorized under the following categories: biosynthetic process (21.43%), carbohydrate metabolic process (14.28%), response to biotic stimulus (10.71%) and response to stress (10.71%). Biosynthetic process had the largest proportion of expressed genes despite having less than 2% of expressed genes in the whole transcriptome analysis. In contrast to the whole transcriptome analysis, carbohydrate metabolic process and cell cycle had larger proportions of expressed genes, whereas response to abiotic stimulus and response to chemicals had smaller proportions of expressed genes. Three of the top five categories were classified under the response to stimulus category.

The 100 most downregulated genes in nickel-treated individuals compared to water controls were annotated and categorized based on metabolic process. They were categorized under the following terms: hydrolase activity (36.36%) and transporter activity (27.27%). These categories had smaller proportions of expressed genes in the whole transcriptome analysis, and hydrolase activity represented less than 2% of expressed genes. The other categories had an equal distribution of genes (Figure 4b).

The 100 most downregulated genes in nickel-treated plants compared to water controls were annotated and categorized based on cellular compartment. They were categorized under the following categories: plasma membrane (41.67%) and extracellular region (33.33%). There were five categories represented, which was lower than in the whole transcriptome analysis, which had 10 or more categories (Figure 4c).

### 3.5. Top Differentially Expressed Genes for Pairwise Comparisons

Genes encoding trypsin inhibitors and cysteine proteinase inhibitors were identified among the top upregulated genes (Table 2 and Table S1). Another identified gene encodes a RING-H2 finger protein, which is involved in the ubiquitin proteasome pathway (Table 1 and Table S1). Several top upregulated genes encode products involved in the jasmonic acid mediated signaling pathway (Table 2 and Table S1).

Genes encoding subtilisin-like proteases were identified among the top downregulated genes (Table 1 and Table S2). Several top downregulated genes encode enzymes involved in the flavonoid biosynthetic process (Table 2 and Table S2). Two genes encoding a probable PIP2-8 aquaporin were identified among the top downregulated genes (Table 1 and Table S2). Genes encoding cellulose synthase A subunits and a gene encoding the WALLS ARE THIN1 (WAT1) protein were identified (Table 2 and Table S2).

## 4. Discussion

### 4.1. Differential Gene Expression (DEG) Analysis

*Pinus banksiana* seedlings exhibited moderate tolerance in response to excess nickel. In metal-contaminated sites in Sudbury, *Pinus banksiana* was found to have moderate genetic diversity and low gene flow, which may have been factors that contributed to its overall heavy metal tolerance [22,27]. *Pinus banksiana* is able to accumulate nickel in the needles, roots and branches, albeit to a lesser extent than would be required to be classified as an accumulator [22,28]. Previous reports have provided differences in gene expression between nickel-treated and -untreated hardwood genotypes [29,30,31]. The greater number of upregulated genes compared to downregulated genes in the present study indicates that excess nickel mostly elicited an increase in protein production.

### 4.2. Gene Ontology of the Top 100 DEGs in Response to Excess Nickel

To further describe the transcriptome in response to excess nickel, analysis of the top differentially expressed genes (DEGs) was used to filter highly regulated mechanisms and processes from those with lower background expression. The top DEGs undergo the greatest amount of expression, thereby serving as reliable indicators of mechanisms that are most likely to be involved in nickel tolerance. Gene ontology of the top DEGs can categorize these processes into discernable functions with interpretive value. The largest proportion of upregulated genes in nickel-treated plants compared to the controls was associated with the response to stress, implicating the prominence of stress mitigation in nickel tolerance (Figure 3a). Commonly reported symptoms of nickel stress include oxidative damage, photoinhibition, loss of water retention, cellular senescence and growth inhibition [7,15,16,32,33,34,35]. Under adverse conditions, processes associated with stress mitigation can counteract symptoms by maintaining the homeostasis of substances, minimizing tissue damage and ensuring the proper functioning of enzymes [36,37,38]. Some stress response mechanisms of excess nickel include the upregulation of antioxidant enzymes, antioxidant production, cell wall thickening and proline accumulation [32,39,40,41,42,43]. Genes that are categorized in response to chemicals, to abiotic stimulus and to biotic stimulus may be linked to the stress response for two distinct reasons. The annotation of the top regulated genes revealed that many of the genes involved in the stress response were multifaceted and functionally related under the same parent term. The large proportion of upregulated genes involved in signal transduction indicated the significance of cellular communication in the mediation of physiological changes [44]. Multiple studies of nickel-afflicted plants have characterized the involvement of signaling in stress mitigation, stress-related crosstalk and growth regulation [45,46,47,48,49,50]. Signaling pathways induced by nickel stress may include auxin, cytokinin and ethylene [51].

The terms with the largest proportions of downregulated genes in nickel-treated plants compared to water controls were associated with the biosynthetic process, the carbohydrate metabolic process and the response to stress (Figure 4a). The biosynthetic process is an expansive category that encompasses numerous products and entities [42,43]. In response to excess nickel, the plant may elicit changes to the biosynthetic process to streamline the production of specific substances to confer higher tolerance. Downregulation of biosynthesis could reduce the production of substances such as ethylene, which has the potential to hinder nickel tolerance and accelerate senescence when produced in excess [51,52]. The synthesis of substances that further exasperate tissue damage under compromised conditions, such as hydrogen peroxide, may also be downregulated to preserve tissue integrity and ensure proper organelle functioning [53]. In response to heavy metals, the downregulation of genes involved in the carbohydrate metabolic process depends on the physiological requirements of the plant. The reduced breakdown of structural polymers, such as cellulose and pectin, has been shown to maintain the strength of the cell wall [54,55]. Conversely, the preservation of constituent monomers and other intermediates may have a role in the regulation of metabolism [56].

Under the same nickel-treatment regimen, the transcriptomes of *Populus tremuloides, Betula papyrifera* and *Acer rubrum* elicited the majority of the gene expression in nickel transport, cellular component organization and the carbohydrate metabolic process [29,30,31]. Gene expression associated with metabolic function was similar among the different species. Unlike the previously mentioned species, the plasma membrane comprised the largest proportion of gene expression for the cellular component term. The plasma membrane is the second layer that interacts with heavy metals and is thus affected by nickel stress. Excess nickel induces the production of malondialdehyde, which causes lipid peroxidation and membrane instability [57]. Receptors, ligands and other intermediates on the plasma membrane may be involved in signal transduction and the response to stress [58,59,60]. Additionally, genes associated with the stress response may be involved in maintaining membrane integrity and preventing electrolyte leakage [33,61]. The small proportion of genes associated with transport indicates that the majority of genes were not associated with nickel transporters (Figure 3 and Figure 4). Unlike *Pinus banksiana*, the transcriptomes of the aforementioned angiosperms had the majority of genes associated with the ribosome, attributed to increased protein translation [29,30,31]. Overall, the large functional differences between the transcriptomes of angiosperms and *Pinus banksiana* demonstrate that *Pinus banksiana* manages excess nickel differently from angiosperms.

### 4.3. Annotation of the Top Upregulated Genes between Nickel-Treated Plants and the Control

GO annotation of the top DEGs in nickel-treated samples compared to water controls could elucidate the function of genes and the molecular mechanisms that differentiate treated plants from untreated plants. Although the annotation of the top 100 genes is informative, it only accounts for a fraction of total expressed genes and is not an exhaustive list that encompasses all highly expressed genes. Trypsin inhibitors and cysteine proteinase inhibitors were upregulated. These proteases downregulate serine protease and cysteine proteinase activity, respectively [62,63]. The upregulation of different proteases is a response to various stressors, such as drought, herbivory and heavy metal toxicity [63,64,65,66,67]. Excess nickel can cause overproduction of ROS, which can damage proteins and cause misfolding, resulting in an increase in protease activity [68,69]. Large amounts of nickel stress can cause decreases in protein content, increases in protein aggregation and unsustainable levels of protein breakdown, which may compromise cell viability [32,70,71,72]. The upregulation of trypsin inhibitors and cysteine proteinase inhibitors could be a counteractive measure to elevated levels of protease activity caused by nickel toxicity. In addition to proteinase inhibition, the cysteine proteinase inhibitor cystatin in *Brassica juncea* has been reported to have the ability to chelate nickel [67].

The RING-H2 finger protein, which was also upregulated, is a E3 ubiquitin ligase that initiates the ubiquitin proteasome pathway by recognizing misfolded or non-functional proteins caused by stressors, such as excess nickel [73,74]. Damaged proteins that are processed though the ubiquitin proteasome system (UPS) pathway are eventually degraded in the proteasome [75]. The UPS can aid in the modulation of stress signaling by regulating the numbers of proteins and transcription factors involved in the stress response [76]. In other plants, increased expression of E3 ubiquitin ligases counteracted heavy metal stress by elevating the expression of antioxidant enzymes, reducing ROS and repressing the transportation of heavy metals via chelation [77,78]. Under high salinity and drought stress, the RING-H2 finger protein can also regulate the synthesis of ABA, which is a hormone involved in stress mitigation and stress-associated signaling [79,80].

Two other identified genes encode TIFY jasmonate ZIM-domain proteins, which actively repress jasmonate signaling unless degraded by the ubiquitin-proteasome pathway [81,82]. Another identified gene encodes a jasmonate-induced oxygenase that negatively regulates jasmonate signaling by converting jasmonate into the inactive conjugate 12-hydroxyjasmonate [83]. Jasmonates are stress-induced hormones that reduce cell replication, cell size and photosynthetic activity in lieu of driving tissue repair and increasing the production of defense molecules, such as jasmonate inducible proteins [45,84]. Some studies have reported the use of jasmonates in the alleviation of heavy metal toxicity, whereas other studies have reported decreased tolerance [85,86,87]. Additionally, studies have also used jasmonate inhibitors to alleviate heavy metal toxicity [88,89]. The culmination of various studies suggests that the physiological effects of genes associated with the jasmonic acid-mediated signaling pathway are dependent on the growth priority and the state of photosynthesis in the plant.

### 4.4. Annotation of the Top Downregulated Genes between Nickel-Treated Plants and the Control

GO annotation of top downregulated genes in nickel-treated plants compared to water controls could characterize genes with reduced expression in the treatment group. Subtilisin-like proteases that were among the top downregulated genes code for serine-type endopeptidases that facilitate the breakdown of peptide bonds using serine as a nucleophilic center [90]. Stressors such as heavy metals and drought cause protein damage and dysfunction, eliciting the response of subtilisin-like proteases [90,91]. Downregulation of subtilisin-like proteases preserves cell viability by reducing the level of protein breakdown and maintaining the proteome. The reduction in protein breakdown prevents the inhibition of various processes that may have occurred were protease activity left unchecked [71,72]. Downregulation of this gene is consistent with the proposed function of the previously described trypsin inhibitor genes, which also inhibit protein breakdown.

One of the identified downregulated genes encodes flavonol synthase, which catalyzes the production of flavonol [92]. Another identified gene encodes chalcone synthase, an enzyme that catalyzes the production of naringenin chalcone, which serves as an initial precursor to flavonoids [93]. Additionally, an identified gene encodes anthocyanidin reductase, which converts anthocyanidin into flavan-3-ol [94,95]. Downregulation of these enzymes reduces the production of flavonoids, with broad impacts on plant physiology and the stress response [93,96,97,98,99]. Under various stressors, flavanols have been implicated in scavenging ROS, regulating auxin levels and improving growth [100,101,102]. Downregulation of flavonoid production could also be a response to dysregulated iron homeostasis caused by nickel toxicity. Excess nickel causes a severe disruption of iron homeostasis by obstructing the initial uptake of iron into the root cells and reducing the iron transportation from the roots to shoots [11,103]. Decreased levels of iron cause the competitive inhibition of photosystem II, diminished chlorophyll function and reduced chlorophyll production [11,104,105]. Flavonols and, to a lesser extent, flavan-3-ols have a high binding affinity to iron [100,106,107,108,109]. The downregulation of iron chelators could increase the availability of iron ions and maintain iron homeostasis, thereby counteracting a prominent symptom of nickel toxicity. In some studies, the flavonol quercetin inhibited iron absorption and uptake in animals [110,111]. The role of flavonols in nickel tolerance has not yet been investigated.

PIP2-8 aquaporins, which were also among the top downregulated protein, are transporters with a broad specificity that transports water and small solutes between cells [112]. Downregulation of aquaporins may be a response to multiple symptoms caused by nickel toxicity, including decreased water content, reduced transpiration and disturbances in metal homeostasis [11,12,33,34]. Decreased aquaporin expression could potentially decrease the intracellular transportation of heavy metals, retain water content and maintain the proper homeostasis of other metals [113,114].

WAT1, which was among the top downregulated proteins, is a vacuolar auxin transporter that exports auxin from the vacuole to the cytoplasm and is an integral component of intracellular auxin homeostasis [115]. Excess nickel can inhibit growth and development by decreasing the distribution of auxin throughout the shoots [116]. Downregulation of WAT1 may exasperate growth inhibition by further reducing intracellular levels of auxin [115,117]. It is also possible that downregulation of WAT1 may elicit an increase in salicylic acid synthesis and signaling, which are involved in various defense pathways [118,119]. In many plants, salicylic acid has been reported to alleviate heavy metal stress by increasing plasma membrane stability, chlorophyll content and antioxidant enzyme activity [120,121,122].

Genes encoding cellulose synthase A subunits were identified among the top downregulated genes and are involved in the synthesis of cellulose [123]. In *Oryza sativa*, silenced cellulose synthase A subunit genes confer cadmium resistance [124]. The authors attributed the cadmium resistance to possible reductions in the thickness and organization of the cell wall and xylem vasculature. Alterations in the morphology of the xylem decreased cadmium accumulation in the xylem sap, thereby reducing the root-to-shoot translocation of cadmium. It is plausible that these physical changes also affect the accumulation of other heavy metals, such as nickel.

## 5. Conclusions

A comprehensive transcriptome analysis of *Pinus banksiana* was performed in response to excess nickel. The gene expression of plants responding to excess nickel was assessed based on various attributes provided by the transcriptome analysis. Nickel-treated plants had 35–51 million sequences. The de novo transcript assembly identified 581,037 transcripts and 435,293 genes. There were 4128 upregulated genes and 3754 downregulated genes in nickel-treated plants compared to the control. The response to stress and response to chemicals terms comprised the highest proportion of upregulated gene expression whereas the biosynthetic process and carbohydrate metabolic process terms had the highest proportion of downregulated gene expression. The majority of upregulated genes were expressed in the extracellular region and the nucleus whereas the majority of downregulated genes were expressed in the plasma membrane and extracellular region. Notable top upregulated and downregulated genes were mostly associated with the stress response and included genes encoding trypsin inhibitors, RING-H2 finger proteins, Jasmonate ZIM-domain proteins, aquaporin proteins, ABA-related proteins and enzymes involved in the flavonoid biosynthetic process. There were no identified genes that encoded nickel transporters or chelators and mechanisms for nickel resistance could not be described. Transcriptome analysis of *Pinus banksiana* was able to provide detailed information on gene expression in response to nickel toxicity.

## Figures and Tables

**Table 1:** Differentially expressed genes from nickel-treated plants compared to the water controls in *Pinus banksiana*.

Cutoff	Standard (Two-Fold and FDR 0.05)	Low Stringency (Two-Fold and *p* Value 0.01)
Upregulated genes	4128	11,903
Downregulated genes	3754	6332
Total genes	7882	18,235

**Table 2:** Top 25 upregulated genes from nickel-treated plants compared to the controls in *Pinus banksiana.* Top 25 downregulated genes from nickel-treated plants compared to the controls in *Pinus banksiana*.

Rank	Gene ID	Res 1	Res 2	Res 3	Water 1	Water 2	Water 3	logFC	Adj. *p* Value	UniProt Description
0	TRINITY_DN2786_c0_g1	767.81	197.57	545.86	0	0	0	13.96	0.00116	Predicted Protein
1	TRINITY_DN5716_c0_g1	2328.59	913.58	3881.87	0	7.03	0.41	13.34	0.00029	Predicted Protein
2	TRINITY_DN57079_c0_g1	339.53	238.75	261.65	0	0	0	13.30	0.00002	Predicted Protein
3	TRINITY_DN5965_c1_g1	1173.34	760.7	1106.06	0.33	0	0	13.28	0.00009	Predicted Protein
4	TRINITY_DN258556_c0_g1	280.75	98.46	494.55	0	0	0	13.09	0.00181	Predicted Protein
5	TRINITY_DN1368_c0_g1	1156.77	736.4	2060.57	0	1.3	0.07	12.99	0.00047	Predicted Protein
6	TRINITY_DN2832_c0_g1	334.2	111.71	258.08	0	0	0	12.93	0.00056	Predicted Protein
7	TRINITY_DN1628_c0_g1	646.38	288.02	710.02	0	0.32	0	12.82	0.00065	Trypsin inhibitor [Cleaved into: Trypsin inhibitor chain A; Trypsin inhibitor chain B]
8	TRINITY_DN7061_c1_g1	158.35	218.82	172.81	0	0	0	12.69	0.00000	Predicted Protein
9	TRINITY_DN690_c0_g1	494.67	136.83	407.74	0.05	0	0	12.50	0.00181	Predicted Protein
10	TRINITY_DN5795_c0_g1	753.52	420.03	412.9	0	0.84	0	12.43	0.00032	Predicted Protein
11	TRINITY_DN1520_c0_g1	398.05	358.51	936.72	0.02	0.65	0	11.81	0.00043	Trypsin inhibitor [Cleaved into: Trypsin inhibitor chain A; Trypsin inhibitor chain B]
12	TRINITY_DN3861_c0_g1	179.52	38.1	108.51	0	0	0	11.70	0.00251	Predicted Protein
13	TRINITY_DN40097_c0_g1	440.62	297.68	1698.09	0	3.39	0.69	11.62	0.00080	Predicted Protein
14	TRINITY_DN2463_c0_g1	301.76	196.16	568.86	0	0.04	0.11	11.56	0.00056	Predicted Protein
15	TRINITY_DN4524_c0_g3	64.05	74.91	115.6	0	0	0	11.54	0.00002	Predicted Protein
16	TRINITY_DN792_c0_g1	149.05	126.17	86.37	0	0.03	0	11.54	0.00004	ACT domain-containing protein ACR4 (Protein ACT DOMAIN REPEATS 4)
17	TRINITY_DN792_c0_g1	149.05	126.17	86.37	0	0.03	0	11.54	0.00004	ACT domain-containing protein ACR5 (Protein ACT DOMAIN REPEATS 5)
18	TRINITY_DN129489_c0_g1	125.97	40.97	102.59	0	0	0	11.53	0.00085	Predicted Protein
19	TRINITY_DN2914_c0_g1	134.07	79.52	144.69	0	0.03	0	11.51	0.00014	Protein TIFY 10b, OsTIFY10b (Jasmonate ZIM domain-containing protein 7, OsJAZ7) (OsJAZ6)
20	TRINITY_DN2914_c0_g1	134.07	79.52	144.69	0	0.03	0	11.51	0.00014	Protein TIFY 3B (Jasmonate ZIM domain-containing protein 12)
21	TRINITY_DN3536_c0_g1	51.58	119.55	84.19	0	0	0	11.51	0.00001	Predicted Protein
22	TRINITY_DN1537_c0_g1	64.53	90.63	76.46	0	0	0	11.44	0.00000	Predicted Protein
23	TRINITY_DN2075_c1_g1	81.81	56.05	84.87	0	0	0	11.38	0.00005	Predicted Protein
24	TRINITY_DN12750_c0_g1	93.87	62.85	64.95	0	0	0	11.37	0.00005	Predicted Protein
25	TRINITY_DN3685_c0_g2	524.13	169.45	298.36	0.01	0.58	0	11.33	0.00171	Copia protein (Gag-int-pol protein) [Cleaved into: Copia VLP protein; Copia protease, EC 3.4.23.]
0	TRINITY_DN1118_c0_g1	0	0	0	27.63	15.12	24.7	−11.36	4.86 × 10^−5^	Flavonol synthase/flavanone 3-hydroxylase, FLS, EC 1.14.11.9, EC 1.14.20.6
1	TRINITY_DN26931_c0_g1	0.16	0	0	65.61	45.82	36.39	−11.19	9.47 × 10^−5^	Probable aquaporin PIP2-8 (Plasma membrane intrinsic protein 2-8, AtPIP2;8) (Plasma membrane intrinsic protein 3b, PIP3b)
2	TRINITY_DN432_c0_g1	0	0.3	0	77.54	17.58	69.88	−11.15	0.002533	Predicted Protein
3	TRINITY_DN4059_c0_g1	0	0	0	20.09	12.1	19.58	−10.99	4.10 × 10^−5^	Predicted Protein
4	TRINITY_DN30654_c0_g1	0	0	0	14.69	11.78	14.4	−10.67	1.63 × 10^−5^	Predicted Protein
5	TRINITY_DN2314_c0_g1	0.03	0.13	0	40.88	14.56	52.04	−10.43	0.001066	Predicted Protein
6	TRINITY_DN69830_c0_g4	0	0	0	10.13	7.29	18.59	−10.38	0.000101	Predicted Protein
7	TRINITY_DN129793_c0_g1	0	0	0	8.28	13.37	9.36	−10.22	9.45 × 10^−6^	Putative UPF0481 protein At3g02645
8	TRINITY_DN40558_c0_g1	0.04	0	0.05	36.31	14.48	19.64	−10.08	0.000432	Predicted Protein
9	TRINITY_DN522_c0_g3	0	0	0	8.24	4.71	17.93	−10.05	0.000408	Predicted Protein
10	TRINITY_DN1550_c0_g1	0	0.07	0	18.5	9.11	17.44	−9.99	0.000209	Predicted Protein
11	TRINITY_DN113586_c0_g1	0	0	0	7.25	5.74	13.28	−9.94	8.70 × 10^−5^	Predicted Protein
12	TRINITY_DN25689_c0_g1	0.06	0.09	0	26.01	16.36	31.07	−9.92	0.000136	Predicted Protein
13	TRINITY_DN26605_c0_g1	0	0	0	6.61	7.11	10.35	−9.87	2.28 × 10^−5^	Predicted Protein
14	TRINITY_DN31123_c0_g2	0	0	0	6.35	8.14	8.67	−9.82	1.28 × 10^−5^	Predicted Protein
15	TRINITY_DN4890_c0_g1	0	0	0.17	15.59	12.05	25.46	−9.82	0.000174	Predicted Protein
16	TRINITY_DN5062_c0_g2	0	0	0	10.4	7.97	3.99	−9.72	0.000193	Predicted Protein
17	TRINITY_DN3390_c0_g1	0	0	0	9.96	3.94	7.77	−9.69	0.000273	Predicted Protein
18	TRINITY_DN6314_c0_g1	0	0	0	7.61	6.68	5.98	−9.66	2.86 × 10^−5^	Predicted Protein
19	TRINITY_DN2507_c0_g1	0	0	0.61	32.43	13.12	23.67	−9.65	0.000952	Predicted Protein
20	TRINITY_DN53932_c0_g1	0.01	0	0.2	17.81	11.58	17.09	−9.61	0.00016	Predicted Protein
21	TRINITY_DN20386_c0_g1	0	0	0	7.77	6	5.13	−9.55	5.04 × 10^−5^	Predicted Protein
22	TRINITY_DN17540_c0_g1	0	0	0	10.32	6.62	3.33	−9.55	0.000363	Predicted Protein
23	TRINITY_DN51950_c1_g1	0	0	0	6.24	5.15	7.26	−9.53	3.46 × 10^−5^	Predicted Protein
24	TRINITY_DN59077_c1_g1	0	0.2	0	11.37	9.17	20.15	−9.52	0.000196	Predicted Protein
25	TRINITY_DN26_c1_g1	0	0	0	5.86	4.64	7.86	−9.49	5.04 × 10^−5^	Alpha-galactosidase, EC 3.2.1.22 (Alpha-D-galactoside galactohydrolase) (Melibiase)

## Data Availability

We have deposited data in the following depository: Repository/DataBank Accession: NCBI; BioProject accession number PRJNA962116; Databank URL: http://www.ncbi.nlm.nih.gov/genbank, (accessed on 1 January 2022); Repository/DataBank Accession: EmbL; BioProject accession number PRJNA962116; Databank URL: http://www.ebi.ac.uk/ena, (accessed on 1 January 2022).

## Supplementary Materials

The following supporting information can be downloaded at: https://www.mdpi.com/article/10.3390/plants12152889/s1, Table S1: Top 100 upregulated genes from nickel treated plants compared to the water controls in *Pinus banksiana*; Table S2: Top 100 downregulated genes from nickel treated plants compared to the control in *Pinus banksiana*; Figure S1: *Pinus banksiana* seedlings water growing in a growth chamber after treatments with nickel sulfate, potassium sulfate, and water.
