# Supplementary material for: Transcriptome Analysis Reveals Changes in Whole Gene Expression, Biological Process, and Molecular Functions Induced by Nickel in Jack Pine (Pinus banksiana)
Source: Plants (Basel). 2023 Aug 7;12(15):2889. doi: 10.3390/plants12152889 (PMC10421529; doi:10.3390/plants12152889)
Supplement: Supplementary file 1 [file plants-12-02889-s001.zip › plants-2435212-supplementary.pdf]

**Table S1.** Top 100 upregulated genes from nickel treated plants compared to the water controls in *Pinus banksiana*

| Rank | Gene ID | Res 1 | Res 2 | Res 3 | Water 1 | Water 2 | Water 3 | logFC | Adj. P. Value | UniProt Description |
|---|---|---|---|---|---|---|---|---|---|---|
| **0** | TRINITY_DN2786_c0_g1 | 767.81 | 197.57 | 545.86 | 0 | 0 | 0 | 13.96 | 0.00116 | Predicted Protein |
| **1** | TRINITY_DN5716_c0_g1 | 2328.59 | 913.58 | 3881.87 | 0 | 7.03 | 0.41 | 13.34 | 0.00029 | Predicted Protein |
| **2** | TRINITY_DN57079_c0_g1 | 339.53 | 238.75 | 261.65 | 0 | 0 | 0 | 13.30 | 0.00002 | Predicted Protein |
| **3** | TRINITY_DN5965_c1_g1 | 1173.34 | 760.7 | 1106.06 | 0.33 | 0 | 0 | 13.28 | 0.00009 | Predicted Protein |
| **4** | TRINITY_DN258556_c0_g1 | 280.75 | 98.46 | 494.55 | 0 | 0 | 0 | 13.09 | 0.00181 | Predicted Protein |
| **5** | TRINITY_DN1368_c0_g1 | 1156.77 | 736.4 | 2060.57 | 0 | 1.3 | 0.07 | 12.99 | 0.00047 | Predicted Protein |
| **6** | TRINITY_DN2832_c0_g1 | 334.2 | 111.71 | 258.08 | 0 | 0 | 0 | 12.93 | 0.00056 | Predicted Protein |
| **7** | TRINITY_DN1628_c0_g1 | 646.38 | 288.02 | 710.02 | 0 | 0.32 | 0 | 12.82 | 0.00065 | Trypsin inhibitor [Cleaved into: Trypsin inhibitor chain A; Trypsin inhibitor chain B ] |
| **8** | TRINITY_DN7061_c1_g1 | 158.35 | 218.82 | 172.81 | 0 | 0 | 0 | 12.69 | 0.00000 | Predicted Protein |
| **9** | TRINITY_DN690_c0_g1 | 494.67 | 136.83 | 407.74 | 0.05 | 0 | 0 | 12.50 | 0.00181 | Predicted Protein |
| **10** | TRINITY_DN5795_c0_g1 | 753.52 | 420.03 | 412.9 | 0 | 0.84 | 0 | 12.43 | 0.00032 | Predicted Protein |
| **11** | TRINITY_DN1520_c0_g1 | 398.05 | 358.51 | 936.72 | 0.02 | 0.65 | 0 | 11.81 | 0.00043 | Trypsin inhibitor [Cleaved into: Trypsin inhibitor chain A; Trypsin inhibitor chain B ] |
| **12** | TRINITY_DN3861_c0_g1 | 179.52 | 38.1 | 108.51 | 0 | 0 | 0 | 11.70 | 0.00251 | Predicted Protein |
| **13** | TRINITY_DN40097_c0_g1 | 440.62 | 297.68 | 1698.09 | 0 | 3.39 | 0.69 | 11.62 | 0.00080 | Predicted Protein |
| **14** | TRINITY_DN2463_c0_g1 | 301.76 | 196.16 | 568.86 | 0 | 0.04 | 0.11 | 11.56 | 0.00056 | Predicted Protein |
| **15** | TRINITY_DN4524_c0_g3 | 64.05 | 74.91 | 115.6 | 0 | 0 | 0 | 11.54 | 0.00002 | Predicted Protein |
| **16** | TRINITY_DN792_c0_g1 | 149.05 | 126.17 | 86.37 | 0 | 0.03 | 0 | 11.54 | 0.00004 | ACT domain-containing protein ACR4 (Protein ACT DOMAIN REPEATS 4) |
| **17** | TRINITY_DN792_c0_g1 | 149.05 | 126.17 | 86.37 | 0 | 0.03 | 0 | 11.54 | 0.00004 | ACT domain-containing protein ACR5 (Protein |

| | | | | | | | | | | |
|---|---|---|---|---|---|---|---|---|---|---|
| | | | | | | | | | | ACT DOMAIN REPEATS 5) |
| **18** | TRINITY_DN129489_c0_g1 | 125.97 | 40.97 | 102.59 | 0 | 0 | 0 | 11.53 | 0.00085 | Predicted Protein |
| **19** | TRINITY_DN2914_c0_g1 | 134.07 | 79.52 | 144.69 | 0 | 0.03 | 0 | 11.51 | 0.00014 | Protein TIFY 10b, OsTIFY10b (Jasmonate ZIM domain-containing protein 7, OsJAZ7) (OsJAZ6) |
| **20** | TRINITY_DN2914_c0_g1 | 134.07 | 79.52 | 144.69 | 0 | 0.03 | 0 | 11.51 | 0.00014 | Protein TIFY 3B (Jasmonate ZIM domain-containing protein 12) |
| **21** | TRINITY_DN3536_c0_g1 | 51.58 | 119.55 | 84.19 | 0 | 0 | 0 | 11.51 | 0.00001 | Predicted Protein |
| **22** | TRINITY_DN1537_c0_g1 | 64.53 | 90.63 | 76.46 | 0 | 0 | 0 | 11.44 | 0.00000 | Predicted Protein |
| **23** | TRINITY_DN2075_c1_g1 | 81.81 | 56.05 | 84.87 | 0 | 0 | 0 | 11.38 | 0.00005 | Predicted Protein |
| **24** | TRINITY_DN12750_c0_g1 | 93.87 | 62.85 | 64.95 | 0 | 0 | 0 | 11.37 | 0.00005 | Predicted Protein |
| **25** | TRINITY_DN3685_c0_g2 | 524.13 | 169.45 | 298.36 | 0.01 | 0.58 | 0 | 11.33 | 0.00171 | Copia protein (Gag-int-pol protein) [Cleaved into: Copia VLP protein; Copia protease, EC 3.4.23.- ] |
| **26** | TRINITY_DN3069_c0_g1 | 310.77 | 133.21 | 112.57 | 0 | 0.37 | 0 | 11.16 | 0.00120 | Predicted Protein |
| **27** | TRINITY_DN4477_c1_g1 | 58.41 | 73.47 | 40.83 | 0 | 0 | 0 | 11.00 | 0.00002 | Predicted Protein |
| **28** | TRINITY_DN9955_c0_g1 | 42.57 | 42.66 | 81.29 | 0 | 0 | 0 | 10.91 | 0.00007 | Predicted Protein |
| **29** | TRINITY_DN3861_c0_g2 | 73.03 | 22.1 | 87.52 | 0 | 0 | 0 | 10.90 | 0.00200 | Predicted Protein |
| **30** | TRINITY_DN2496_c0_g1 | 164.42 | 56.24 | 74.78 | 0 | 0.08 | 0 | 10.89 | 0.00111 | Predicted Protein |
| **31** | TRINITY_DN3195_c0_g2 | 97.14 | 27.64 | 44.27 | 0 | 0 | 0 | 10.82 | 0.00125 | Predicted Protein |
| **32** | TRINITY_DN13148_c0_g2 | 149.44 | 53.58 | 71 | 0.03 | 0 | 0 | 10.80 | 0.00087 | Predicted Protein |
| **33** | TRINITY_DN8563_c1_g1 | 81.63 | 24.89 | 51.8 | 0 | 0 | 0 | 10.76 | 0.00105 | Predicted Protein |
| **34** | TRINITY_DN4828_c0_g1 | 77.75 | 87.05 | 75 | 0 | 0.07 | 0 | 10.73 | 0.00002 | Predicted Protein |
| **35** | TRINITY_DN1453_c1_g4 | 164.09 | 61.99 | 144.64 | 0 | 0.3 | 0 | 10.72 | 0.00143 | Predicted Protein |
| **36** | TRINITY_DN1031_c0_g1 | 92.79 | 147.67 | 132.23 | 0 | 0.42 | 0 | 10.65 | 0.00004 | Putative cysteine-rich repeat secretory protein 17 |

| | | | | | | | | | | |
|---|---|---|---|---|---|---|---|---|---|---|
| **37** | TRINITY_DN17_c0_g2 | 50.63 | 58.32 | 26.97 | 0 | 0 | 0 | 10.62 | 0.00006 | RING-H2 finger protein ATL60, EC 2.3.2.27 (RING-type E3 ubiquitin transferase ATL60) |
| **38** | TRINITY_DN1299_c1_g1 | 32.6 | 35.97 | 67.12 | 0 | 0 | 0 | 10.60 | 0.00007 | Predicted Protein |
| **39** | TRINITY_DN2516_c0_g1 | 445.31 | 249.4 | 199.81 | 0.02 | 0.63 | 0.07 | 10.58 | 0.00032 | Predicted Protein |
| **40** | TRINITY_DN1518_c0_g1 | 96.36 | 25.43 | 28.14 | 0 | 0 | 0 | 10.56 | 0.00213 | Predicted Protein |
| **41** | TRINITY_DN678_c0_g1 | 132.68 | 116.88 | 131.13 | 0 | 0.61 | 0 | 10.55 | 0.00012 | Cysteine proteinase inhibitor 6, AtCYS-6 (PIP-M) (PRLI-interacting factor M) |
| **42** | TRINITY_DN678_c0_g1 | 132.68 | 116.88 | 131.13 | 0 | 0.61 | 0 | 10.55 | 0.00012 | Cysteine proteinase inhibitor 3, AtCYS-3 |
| **43** | TRINITY_DN678_c0_g1 | 132.68 | 116.88 | 131.13 | 0 | 0.61 | 0 | 10.55 | 0.00012 | Multicystatin, MC |
| **44** | TRINITY_DN125473_c0_g2 | 65.02 | 24.25 | 42.52 | 0 | 0 | 0 | 10.54 | 0.00058 | Predicted Protein |
| **45** | TRINITY_DN103008_c0_g1 | 216.81 | 147.31 | 109.01 | 0.08 | 0.1 | 0 | 10.48 | 0.00022 | Predicted Protein |
| **46** | TRINITY_DN6089_c0_g1 | 96.41 | 139.06 | 110.48 | 0 | 0.52 | 0 | 10.47 | 0.00004 | Predicted Protein |
| **47** | TRINITY_DN630_c0_g1 | 70.97 | 43.91 | 146.42 | 0 | 0.16 | 0 | 10.40 | 0.00127 | Protein SRG1, AtSRG1 (Protein SENESCENCE-RELATED GENE 1) |
| **48** | TRINITY_DN630_c0_g1 | 70.97 | 43.91 | 146.42 | 0 | 0.16 | 0 | 10.40 | 0.00127 | Jasmonate-induced oxygenase 4, EC 1.14.11.- (2-oxoglutarate-dependent dioxygenase JOX4) (Anthocyanidin synthase) (Jasmonic acid oxidase 4) |
| **49** | TRINITY_DN630_c0_g1 | 70.97 | 43.91 | 146.42 | 0 | 0.16 | 0 | 10.40 | 0.00127 | Codeine O-demethylase, EC 1.14.11.32 |
| **50** | TRINITY_DN630_c0_g1 | 70.97 | 43.91 | 146.42 | 0 | 0.16 | 0 | 10.40 | 0.00127 | S-norcoclaurine synthase 1, CjNCS1, EC 4.2.1.78 |
| **51** | TRINITY_DN1958_c0_g1 | 34.95 | 25.92 | 54.49 | 0 | 0 | 0 | 10.38 | 0.00015 | Predicted Protein |
| **52** | TRINITY_DN3889_c0_g1 | 414.11 | 167.54 | 300.23 | 0 | 0.98 | 0.57 | 10.37 | 0.00068 | Predicted Protein |

| | | | | | | | | | | |
|---|---|---|---|---|---|---|---|---|---|---|
| **53** | TRINITY_DN4195_c0_g1 | 47.94 | 20.18 | 47.91 | 0 | 0 | 0 | 10.36 | 0.00056 | Predicted Protein |
| **54** | TRINITY_DN50999_c1_g1 | 44.58 | 72.78 | 14 | 0 | 0 | 0 | 10.35 | 0.00092 | Predicted Protein |
| **55** | TRINITY_DN5723_c0_g1 | 147.25 | 109.75 | 89.9 | 0.01 | 0.19 | 0 | 10.34 | 0.00014 | Predicted Protein |
| **56** | TRINITY_DN4424_c0_g1 | 47.33 | 16.14 | 56.98 | 0 | 0 | 0 | 10.33 | 0.00157 | Predicted Protein |
| **57** | TRINITY_DN1307_c0_g1 | 16.43 | 96.5 | 26.64 | 0 | 0 | 0 | 10.30 | 0.00055 | Germin-like protein 1-1 (Germin-like protein 4, OsGER4) |
| **58** | TRINITY_DN1307_c0_g1 | 16.43 | 96.5 | 26.64 | 0 | 0 | 0 | 10.30 | 0.00055 | Germin-like protein subfamily 2 member 2 |
| **59** | TRINITY_DN8008_c0_g1 | 41.17 | 24.54 | 37.72 | 0 | 0 | 0 | 10.26 | 0.00013 | Predicted Protein |
| **60** | TRINITY_DN5391_c1_g1 | 39.5 | 79.43 | 11.5 | 0 | 0 | 0 | 10.24 | 0.00179 | Predicted Protein |
| **61** | TRINITY_DN5136_c0_g1 | 45.05 | 28.07 | 26.97 | 0 | 0 | 0 | 10.21 | 0.00012 | Predicted Protein |
| **62** | TRINITY_DN71807_c0_g1 | 29.61 | 23.8 | 49.04 | 0 | 0 | 0 | 10.21 | 0.00014 | Predicted Protein |
| **63** | TRINITY_DN10435_c0_g1 | 688.04 | 977.43 | 155.25 | 0.18 | 0.41 | 0.11 | 10.17 | 0.00093 | Glucan endo-1,3-beta-glucosidase, acidic isoform, EC 3.2.1.39 ((1->3)-beta-glucan endohydrolase, (1->3)-beta-glucanase) (Beta-1,3-endoglucanase) |
| **64** | TRINITY_DN1644_c0_g1 | 42.57 | 17.5 | 40.38 | 0 | 0 | 0 | 10.15 | 0.00061 | NAC transcription factor 47 (NAC domain-containing protein 47, ANAC047) (Protein SPEEDY HYPONASTIC GROWTH) |
| **65** | TRINITY_DN1472_c0_g1 | 212.32 | 210.54 | 174.12 | 0.02 | 0.16 | 0.1 | 10.15 | 0.00002 | Triacylglycerol lipase OBL1, EC 3.1.1.- (Oil body lipase 1, NtOBL1) |
| **66** | TRINITY_DN8703_c1_g1 | 20.94 | 68.98 | 20.74 | 0 | 0 | 0 | 10.14 | 0.00014 | Predicted Protein |
| **67** | TRINITY_DN30360_c0_g2 | 32.32 | 41.67 | 19.9 | 0 | 0 | 0 | 10.09 | 0.00004 | Predicted Protein |
| **68** | TRINITY_DN690_c1_g1 | 36.04 | 15 | 46.52 | 0 | 0 | 0 | 10.06 | 0.00098 | Predicted Protein |
| **69** | TRINITY_DN2595_c0_g1 | 34.43 | 14.93 | 48.4 | 0 | 0 | 0 | 10.06 | 0.00100 | Predicted Protein |

| | | | | | | | | | | |
|---|---|---|---|---|---|---|---|---|---|---|
| **70** | TRINITY_DN5044_c1_g1 | 3556.6 | 1665.87 | 4959.49 | 2.92 | 5.34 | 0.16 | 10.06 | 0.00002 | Predicted Protein |
| **71** | TRINITY_DN2691_c0_g1 | 93.28 | 347.62 | 74.23 | 0 | 0.14 | 0.33 | 10.04 | 0.00108 | Predicted Protein |
| **72** | TRINITY_DN59057_c0_g1 | 979.45 | 270.63 | 1164.92 | 0.55 | 2.6 | 0 | 10.03 | 0.00106 | Predicted Protein |
| **73** | TRINITY_DN5965_c0_g1 | 159.21 | 70.92 | 203.21 | 0.07 | 0.21 | 0 | 10.00 | 0.00161 | Predicted Protein |
| **74** | TRINITY_DN7685_c0_g1 | 69.15 | 75.78 | 88.19 | 0 | 0.41 | 0 | 9.99 | 0.00010 | Predicted Protein |
| **75** | TRINITY_DN25430_c0_g1 | 14.61 | 23.03 | 66.45 | 0 | 0 | 0 | 9.99 | 0.00091 | Predicted Protein |
| **76** | TRINITY_DN1507_c0_g1 | 26.74 | 27.5 | 28.46 | 0 | 0 | 0 | 9.97 | 0.00002 | Predicted Protein |
| **77** | TRINITY_DN183161_c0_g1 | 38.89 | 15.66 | 33.33 | 0 | 0 | 0 | 9.96 | 0.00062 | Predicted Protein |
| **78** | TRINITY_DN2540_c0_g1 | 65.96 | 51.15 | 17.44 | 0 | 0.05 | 0 | 9.95 | 0.00116 | Predicted Protein |
| **79** | TRINITY_DN257933_c1_g1 | 22.27 | 33.69 | 26.6 | 0 | 0 | 0 | 9.95 | 0.00001 | Predicted Protein |
| **80** | TRINITY_DN15707_c0_g1 | 40.5 | 12.74 | 34.71 | 0 | 0 | 0 | 9.90 | 0.00145 | Predicted Protein |
| **81** | TRINITY_DN122303_c0_g2 | 43.54 | 23.48 | 82.74 | 0 | 0 | 0.06 | 9.87 | 0.00149 | Predicted Protein |
| **82** | TRINITY_DN12875_c0_g1 | 368 | 113.74 | 248.93 | 0.24 | 0.34 | 0 | 9.86 | 0.00219 | Predicted Protein |
| **83** | TRINITY_DN157113_c2_g1 | 33.92 | 29.87 | 15.92 | 0 | 0 | 0 | 9.85 | 0.00013 | Predicted Protein |
| **84** | TRINITY_DN6211_c0_g1 | 26.02 | 66.59 | 9.11 | 0 | 0 | 0 | 9.84 | 0.00186 | Predicted Protein |
| **85** | TRINITY_DN5240_c1_g1 | 75.64 | 57.88 | 47.63 | 0 | 0.09 | 0.03 | 9.78 | 0.00014 | Predicted Protein |
| **86** | TRINITY_DN2454_c0_g2 | 24.25 | 22.33 | 25.85 | 0 | 0 | 0 | 9.78 | 0.00003 | Predicted Protein |
| **87** | TRINITY_DN1456_c0_g1 | 306.48 | 253.1 | 155.47 | 0.27 | 0 | 0.25 | 9.78 | 0.00018 | Predicted Protein |
| **88** | TRINITY_DN26886_c0_g1 | 61.81 | 26.91 | 40.92 | 0 | 0.09 | 0 | 9.77 | 0.00069 | Predicted Protein |
| **89** | TRINITY_DN8619_c0_g1 | 37.8 | 12.68 | 28.14 | 0 | 0 | 0 | 9.77 | 0.00109 | Predicted Protein |
| **90** | TRINITY_DN27427_c0_g1 | 48.32 | 16.08 | 14.95 | 0 | 0 | 0 | 9.71 | 0.00155 | Predicted Protein |
| **91** | TRINITY_DN5616_c1_g1 | 114.68 | 86.52 | 56.74 | 0 | 1.17 | 0 | 9.70 | 0.00072 | Predicted Protein |
| **92** | TRINITY_DN21893_c1_g1 | 11.63 | 44.29 | 22.13 | 0 | 0 | 0 | 9.67 | 0.00012 | Predicted Protein |
| **93** | TRINITY_DN7420_c0_g1 | 25.09 | 11.85 | 36.81 | 0 | 0 | 0 | 9.66 | 0.00090 | Predicted Protein |
| **94** | TRINITY_DN49749_c0_g1 | 27.49 | 17.43 | 20.17 | 0 | 0 | 0 | 9.61 | 0.00013 | Predicted Protein |
| **95** | TRINITY_DN5340_c0_g1 | 27.6 | 47.12 | 22.31 | 0 | 0.04 | 0 | 9.60 | 0.00005 | Predicted Protein |
| **96** | TRINITY_DN3840_c0_g2 | 40.87 | 29.93 | 22.16 | 0 | 0.03 | 0 | 9.58 | 0.00017 | Predicted Protein |
| **97** | TRINITY_DN237688_c0_g1 | 21.17 | 36.54 | 11.77 | 0 | 0 | 0 | 9.57 | 0.00015 | Predicted Protein |
| **98** | TRINITY_DN251401_c0_g1 | 26.95 | 28.1 | 11.66 | 0 | 0 | 0 | 9.57 | 0.00020 | Predicted Protein |
| **99** | TRINITY_DN104547_c0_g1 | 238.4 | 83.01 | 249.46 | 0.25 | 0.3 | 0 | 9.56 | 0.00258 | Predicted Protein |

| | | | | | | | | | | |
|---|---|---|---|---|---|---|---|---|---|---|
| **100** | TRINITY_DN395_c0_g1 | 33.63 | 33.79 | 22.39 | 0.02 | 0 | 0 | 9.54 | 0.00005 | Inositol polyphosphate 5-phosphatase OCRL, EC 3.1.3.36, EC 3.1.3.56 (Inositol polyphosphate 5-phosphatase OCRL-1) (Phosphatidylinositol 3,4,5-triphosphate 5-phosphatase, EC 3.1.3.86) |

**Table S2.** Top 100 downregulated genes from nickel treated plants compared to the control in *Pinus banksiana*

| Rank | Gene ID | Res 1 | Res 2 | Res 3 | Water 1 | Water 2 | Water 3 | Adj. P. Value | Protein Description |
|---|---|---|---|---|---|---|---|---|---|
| **0** | TRINITY_DN1118_c0_g1 | 0 | 0 | 0 | 27.63 | 15.12 | 24.7 | 4.86E-05 | Flavonol synthase/flavanone 3-hydroxylase, FLS, EC 1.14.11.9, EC 1.14.20.6 |
| **1** | TRINITY_DN26931_c0_g1 | 0.16 | 0 | 0 | 65.61 | 45.82 | 36.39 | 9.47E-05 | Probable aquaporin PIP2-8 (Plasma membrane intrinsic protein 2-8, AtPIP2;8) (Plasma membrane intrinsic protein 3b, PIP3b) |
| **2** | TRINITY_DN432_c0_g1 | 0 | 0.3 | 0 | 77.54 | 17.58 | 69.88 | 0.002533 | Predicted Protein |
| **3** | TRINITY_DN4059_c0_g1 | 0 | 0 | 0 | 20.09 | 12.1 | 19.58 | 4.10E-05 | Predicted Protein |
| **4** | TRINITY_DN30654_c0_g1 | 0 | 0 | 0 | 14.69 | 11.78 | 14.4 | 1.63E-05 | Predicted Protein |
| **5** | TRINITY_DN2314_c0_g1 | 0.03 | 0.13 | 0 | 40.88 | 14.56 | 52.04 | 0.001066 | Predicted Protein |
| **6** | TRINITY_DN69830_c0_g4 | 0 | 0 | 0 | 10.13 | 7.29 | 18.59 | 0.000101 | Predicted Protein |
| **7** | TRINITY_DN129793_c0_g1 | 0 | 0 | 0 | 8.28 | 13.37 | 9.36 | 9.45E-06 | Putative UPF0481 protein At3g02645 |
| **8** | TRINITY_DN40558_c0_g1 | 0.04 | 0 | 0.05 | 36.31 | 14.48 | 19.64 | 0.000432 | Predicted Protein |
| **9** | TRINITY_DN522_c0_g3 | 0 | 0 | 0 | 8.24 | 4.71 | 17.93 | 0.000408 | Predicted Protein |
| **10** | TRINITY_DN1550_c0_g1 | 0 | 0.07 | 0 | 18.5 | 9.11 | 17.44 | 0.000209 | Predicted Protein |
| **11** | TRINITY_DN113586_c0_g1 | 0 | 0 | 0 | 7.25 | 5.74 | 13.28 | 8.70E-05 | Predicted Protein |
| **12** | TRINITY_DN25689_c0_g1 | 0.06 | 0.09 | 0 | 26.01 | 16.36 | 31.07 | 0.000136 | Predicted Protein |

| | | | | | | | | | |
|---|---|---|---|---|---|---|---|---|---|
| **13** | TRINITY_DN26605_c0_g1 | 0 | 0 | 0 | 6.61 | 7.11 | 10.35 | 2.28E-05 | Predicted Protein |
| **14** | TRINITY_DN31123_c0_g2 | 0 | 0 | 0 | 6.35 | 8.14 | 8.67 | 1.28E-05 | Predicted Protein |
| **15** | TRINITY_DN4890_c0_g1 | 0 | 0 | 0.17 | 15.59 | 12.05 | 25.46 | 0.000174 | Predicted Protein |
| **16** | TRINITY_DN5062_c0_g2 | 0 | 0 | 0 | 10.4 | 7.97 | 3.99 | 0.000193 | Predicted Protein |
| **17** | TRINITY_DN3390_c0_g1 | 0 | 0 | 0 | 9.96 | 3.94 | 7.77 | 0.000273 | Predicted Protein |
| **18** | TRINITY_DN6314_c0_g1 | 0 | 0 | 0 | 7.61 | 6.68 | 5.98 | 2.86E-05 | Predicted Protein |
| **19** | TRINITY_DN2507_c0_g1 | 0 | 0 | 0.61 | 32.43 | 13.12 | 23.67 | 0.000952 | Predicted Protein |
| **20** | TRINITY_DN53932_c0_g1 | 0.01 | 0 | 0.2 | 17.81 | 11.58 | 17.09 | 0.00016 | Predicted Protein |
| **21** | TRINITY_DN20386_c0_g1 | 0 | 0 | 0 | 7.77 | 6 | 5.13 | 5.04E-05 | Predicted Protein |
| **22** | TRINITY_DN17540_c0_g1 | 0 | 0 | 0 | 10.32 | 6.62 | 3.33 | 0.000363 | Predicted Protein |
| **23** | TRINITY_DN51950_c1_g1 | 0 | 0 | 0 | 6.24 | 5.15 | 7.26 | 3.46E-05 | Predicted Protein |
| **24** | TRINITY_DN59077_c1_g1 | 0 | 0.2 | 0 | 11.37 | 9.17 | 20.15 | 0.000196 | Predicted Protein |
| **25** | TRINITY_DN26_c1_g1 | 0 | 0 | 0 | 5.86 | 4.64 | 7.86 | 5.04E-05 | Alpha-galactosidase, EC 3.2.1.22 (Alpha-D-galactoside galactohydrolase) (Melibiase) |
| **26** | TRINITY_DN3304_c0_g1 | 0 | 0 | 0 | 9.07 | 9.08 | 2.36 | 0.000767 | Predicted Protein |
| **27** | TRINITY_DN229927_c0_g1 | 0 | 0 | 0 | 7.04 | 3.93 | 6.56 | 0.000106 | Predicted Protein |
| **28** | TRINITY_DN44526_c0_g2 | 0 | 0 | 0 | 8.45 | 2.63 | 7.68 | 0.000697 | Predicted Protein |
| **29** | TRINITY_DN185135_c0_g1 | 0.01 | 0.04 | 0 | 10.52 | 6.31 | 8.09 | 0.00011 | Predicted Protein |
| **30** | TRINITY_DN69346_c0_g1 | 0 | 0 | 0.11 | 8.89 | 9.05 | 16.12 | 0.000102 | Predicted Protein |
| **31** | TRINITY_DN61932_c0_g1 | 0 | 0 | 0 | 4.25 | 10.03 | 4.53 | 4.75E-05 | Predicted Protein |
| **32** | TRINITY_DN1400_c0_g1 | 0.03 | 0.03 | 0.07 | 24.93 | 11.67 | 26.83 | 0.000432 | Subtilisin-like protease SBT5.6, EC 3.4.21.- (Subtilase subfamily 5 member 6, AtSBT5.6) |
| **33** | TRINITY_DN6996_c0_g5 | 0 | 0.12 | 0 | 12.87 | 8.76 | 10.4 | 0.000112 | Predicted Protein |
| **34** | TRINITY_DN28592_c2_g1 | 0 | 1.27 | 0 | 25.1 | 13.73 | 26.65 | 0.000766 | Predicted Protein |
| **35** | TRINITY_DN63981_c0_g2 | 0 | 0 | 0 | 4.48 | 4.77 | 7.31 | 3.70E-05 | Predicted Protein |
| **36** | TRINITY_DN129749_c0_g1 | 0 | 0.12 | 0 | 9.6 | 9.98 | 11.12 | 4.47E-05 | Predicted Protein |
| **37** | TRINITY_DN800_c0_g2 | 0 | 0 | 0 | 8 | 2.45 | 6.55 | 0.000689 | Predicted Protein |
| **38** | TRINITY_DN1269_c0_g1 | 0.15 | 0.2 | 0 | 32.25 | 13.5 | 37.33 | 0.001037 | Predicted Protein |
| **39** | TRINITY_DN11362_c0_g1 | 0 | 0.89 | 0.23 | 39.53 | 30.88 | 57.71 | 0.000201 | Predicted Protein |

| | | | | | | | | | |
|---|---|---|---|---|---|---|---|---|---|
| **40** | TRINITY_DN20766_c0_g1 | 0 | 0.07 | 0 | 10.17 | 5.57 | 10.32 | 0.000218 | Subtilisin-like protease SBT1.7, EC 3.4.21.- (Cucumisin-like serine protease) (Subtilase subfamily 1 member 7, AtSBT1.7) (Subtilisin-like serine protease 1, At-SLP1) |
| **41** | TRINITY_DN15047_c0_g1 | 0 | 0 | 0 | 4.32 | 5.55 | 5.12 | 1.96E-05 | Predicted Protein |
| **42** | TRINITY_DN26605_c0_g2 | 0 | 0 | 0 | 2.45 | 6.9 | 7.85 | 0.00015 | Predicted Protein |
| **43** | TRINITY_DN104952_c0_g1 | 0 | 0 | 0 | 6.71 | 2.45 | 6.55 | 0.000484 | Predicted Protein |
| **44** | TRINITY_DN4176_c0_g1 | 0.02 | 0.33 | 0.26 | 61.56 | 18.42 | 66.16 | 0.001525 | Chalcone synthase, EC 2.3.1.74 (Naringenin-chalcone synthase) |
| **45** | TRINITY_DN24969_c0_g1 | 0.12 | 0 | 0 | 15.57 | 5.1 | 13.9 | 0.00131 | Predicted Protein |
| **46** | TRINITY_DN24626_c0_g1 | 0.12 | 0.13 | 0 | 21.48 | 21.87 | 17.99 | 6.53E-05 | Predicted Protein |
| **47** | TRINITY_DN121_c0_g3 | 0.09 | 0.02 | 0.11 | 16.6 | 18.32 | 21.49 | 5.20E-05 | Cellulose synthase A catalytic subunit 4 [UDP-forming], AtCesA4, EC 2.4.1.12 (Protein IRREGULAR XYLEM 5, AtIRX5) |
| **48** | TRINITY_DN20218_c0_g1 | 0.02 | 0.04 | 0 | 9.8 | 8.24 | 11.28 | 5.62E-05 | Predicted Protein |
| **49** | TRINITY_DN5372_c0_g1 | 0 | 0 | 0 | 7.25 | 1.67 | 7.18 | 0.001777 | Predicted Protein |
| **50** | TRINITY_DN3173_c0_g2 | 0 | 0 | 0 | 5.65 | 2.3 | 7.09 | 0.000497 | Predicted Protein |
| **51** | TRINITY_DN15841_c0_g1 | 0 | 0 | 0 | 5.23 | 2.71 | 6.29 | 0.000214 | Predicted Protein |
| **52** | TRINITY_DN1891_c0_g3 | 0 | 0 | 0 | 6.76 | 3.67 | 3.42 | 0.000191 | Predicted Protein |
| **53** | TRINITY_DN15910_c0_g1 | 0.1 | 0.24 | 0.09 | 49.33 | 33.41 | 37.21 | 4.83E-05 | Predicted Protein |
| **54** | TRINITY_DN7784_c1_g1 | 0 | 0 | 0 | 8.87 | 2.18 | 3.94 | 0.001207 | Predicted Protein |
| **55** | TRINITY_DN8038_c0_g1 | 0.62 | 0.26 | 0.25 | 122.65 | 81.13 | 105.38 | 4.77E-06 | Probable aquaporin PIP2-8 (Plasma membrane intrinsic protein 2-8, AtPIP2;8) (Plasma membrane intrinsic protein 3b, PIP3b) |
| **56** | TRINITY_DN12836_c0_g1 | 0 | 0 | 0 | 7 | 3.29 | 3.35 | 0.00028 | Alpha carbonic anhydrase 7, AtaCA7, AtalphaCA7, EC 4.2.1.1 (Alpha carbonate dehydratase 7) |
| **57** | TRINITY_DN6386_c0_g2 | 0 | 0 | 0 | 4.88 | 3.66 | 4.48 | 5.51E-05 | Predicted Protein |
| **58** | TRINITY_DN7751_c0_g1 | 0 | 0 | 0 | 5.77 | 2.52 | 5.12 | 0.000267 | Predicted Protein |
| **59** | TRINITY_DN159567_c0_g1 | 0 | 0.32 | 0 | 11.89 | 6.31 | 15.47 | 0.000621 | WAT1-related protein At5g07050 |
| **60** | TRINITY_DN293_c0_g1 | 0.59 | 0.32 | 0.44 | 83.3 | 131.42 | 164.86 | 4.44E-07 | Delta-selinene-like synthase, chloroplastic, PsTPS-SelI, EC 4.2.3.76 |

| | | | | | | | | | |
|---|---|---|---|---|---|---|---|---|---|
| **61** | TRINITY_DN293_c0_g1 | 0.59 | 0.32 | 0.44 | 83.3 | 131.42 | 164.86 | 4.44E-07 | Alpha-humulene synthase, EC 4.2.3.104 (Terpene synthase TPS-Hum, PgTPS-Hum) |
| **62** | TRINITY_DN293_c0_g1 | 0.59 | 0.32 | 0.44 | 83.3 | 131.42 | 164.86 | 4.44E-07 | Delta-selinene synthase, EC 4.2.3.71, EC 4.2.3.76 (Agfdsel1) |
| **63** | TRINITY_DN44886_c0_g1 | 0 | 0.04 | 0 | 6.47 | 4.31 | 7.89 | 0.000134 | Predicted Protein |
| **64** | TRINITY_DN71967_c0_g1 | 0 | 0 | 0 | 6.52 | 2.32 | 4.44 | 0.00046 | Predicted Protein |
| **65** | TRINITY_DN87537_c1_g2 | 0 | 0 | 0 | 4.36 | 1.76 | 7.92 | 0.000982 | Predicted Protein |
| **66** | TRINITY_DN63391_c0_g1 | 0 | 0 | 0 | 6.17 | 1.74 | 5.13 | 0.000994 | Predicted Protein |
| **67** | TRINITY_DN50988_c0_g1 | 0.05 | 0 | 0 | 8.96 | 3.64 | 8.45 | 0.000593 | Predicted Protein |
| **68** | TRINITY_DN36314_c0_g2 | 0 | 0 | 0 | 7.73 | 4.68 | 1.42 | 0.001863 | Putative anthocyanidin reductase, GbANR, EC 1.3.1.- |
| **69** | TRINITY_DN647_c0_g2 | 0 | 0.07 | 0.06 | 15.17 | 6.47 | 13.72 | 0.00068 | Purple acid phosphatase 3, EC 3.1.3.2 |
| **70** | TRINITY_DN256198_c0_g1 | 0 | 0 | 0 | 7.86 | 4.47 | 1.34 | 0.002153 | Predicted Protein |
| **71** | TRINITY_DN125084_c0_g3 | 0.03 | 0 | 0 | 5.87 | 7.75 | 3.36 | 0.000138 | Delta-selinene synthase, EC 4.2.3.71, EC 4.2.3.76 (Agfdsel1) |
| **72** | TRINITY_DN22583_c0_g1 | 0 | 0 | 0 | 4.49 | 3.11 | 3.47 | 8.47E-05 | Predicted Protein |
| **73** | TRINITY_DN98979_c0_g4 | 0 | 0 | 0 | 3.3 | 4.07 | 3.81 | 2.91E-05 | Probable galactinol--sucrose galactosyltransferase 6, EC 2.4.1.82 (Protein DARK INDUCIBLE 10) (Raffinose synthase 6) |
| **74** | TRINITY_DN9649_c1_g3 | 0 | 0 | 0 | 2.73 | 10.46 | 1.9 | 0.000761 | Predicted Protein |
| **75** | TRINITY_DN11419_c0_g1 | 0 | 0 | 0 | 5.05 | 1.36 | 6.38 | 0.001738 | Predicted Protein |
| **76** | TRINITY_DN1911_c0_g1 | 0 | 0.03 | 0.03 | 7.9 | 7.77 | 6.89 | 5.67E-05 | Predicted Protein |
| **77** | TRINITY_DN2236_c0_g1 | 0.2 | 0 | 0.25 | 39.44 | 18.18 | 16.02 | 0.001027 | Predicted Protein |
| **78** | TRINITY_DN250708_c0_g1 | 0 | 0 | 0 | 7.84 | 2 | 2.38 | 0.001663 | Predicted Protein |
| **79** | TRINITY_DN18490_c0_g1 | 0 | 0 | 0 | 3.72 | 1.31 | 8 | 0.002018 | Predicted Protein |
| **80** | TRINITY_DN7878_c0_g1 | 0.25 | 1.75 | 0.46 | 124.44 | 78.86 | 175.53 | 2.73E-06 | Predicted Protein |
| **81** | TRINITY_DN3227_c0_g1 | 0 | 0 | 0 | 3.03 | 2.62 | 5.23 | 0.000109 | Predicted Protein |
| **82** | TRINITY_DN83526_c0_g3 | 0 | 0 | 0 | 2.83 | 5.13 | 3 | 4.20E-05 | Predicted Protein |
| **83** | TRINITY_DN1934_c0_g1 | 0.02 | 0.03 | 0.06 | 16.23 | 6.2 | 17.34 | 0.001139 | Predicted Protein |
| **84** | TRINITY_DN121_c0_g1 | 0.18 | 0.1 | 0.03 | 20.19 | 22.8 | 24.32 | 2.97E-05 | Cellulose synthase A catalytic subunit 8 [UDP-forming], AtCesA8, EC 2.4.1.12 |

| | | | | | | | | | |
|---|---|---|---|---|---|---|---|---|---|
| | | | | | | | | | (Protein IRREGULAR XYLEM 1, AtIRX1) (Protein LEAF WILTING 2) |
| **85** | TRINITY_DN121_c0_g1 | 0.18 | 0.1 | 0.03 | 20.19 | 22.8 | 24.32 | 2.97E-05 | Cellulose synthase A catalytic subunit 9 [UDP-forming], EC 2.4.1.12 (OsCesA9) |
| **86** | TRINITY_DN2085_c0_g1 | 0.34 | 0.57 | 0.14 | 100.49 | 52.63 | 54.79 | 3.54E-05 | Predicted Protein |
| **87** | TRINITY_DN41388_c0_g2 | 0 | 0 | 0.04 | 3.85 | 3.14 | 9.7 | 0.000494 | Predicted Protein |
| **88** | TRINITY_DN17036_c0_g1 | 0.06 | 0.77 | 0.19 | 41.49 | 43.27 | 52.52 | 1.13E-05 | Predicted Protein |
| **89** | TRINITY_DN19058_c0_g1 | 0 | 0 | 0 | 2.83 | 3.41 | 3.9 | 3.74E-05 | Predicted Protein |
| **90** | TRINITY_DN5226_c2_g1 | 0 | 0 | 0 | 3.04 | 1.9 | 6.31 | 0.000435 | Predicted Protein |
| **91** | TRINITY_DN61279_c2_g1 | 0 | 0 | 0 | 5.99 | 1.98 | 2.71 | 0.000712 | Probable LRR receptor-like serine/threonine-protein kinase At5g48740, EC 2.7.11.1 |
| **92** | TRINITY_DN1794_c0_g3 | 0 | 0 | 0 | 2.16 | 2.75 | 6.48 | 0.000243 | Predicted Protein |
| **93** | TRINITY_DN31192_c0_g2 | 0 | 0 | 0 | 3.24 | 3.3 | 3.39 | 4.09E-05 | Predicted Protein |
| **94** | TRINITY_DN260195_c2_g2 | 0 | 0 | 0 | 3.29 | 3.18 | 3.44 | 4.45E-05 | Predicted Protein |
| **95** | TRINITY_DN304016_c0_g1 | 0 | 0 | 0 | 3.72 | 2.27 | 4.05 | 0.000138 | Predicted Protein |
| **96** | TRINITY_DN4245_c0_g1 | 0.06 | 0.24 | 0 | 6.69 | 11.32 | 27.52 | 0.00114 | Predicted Protein |
| **97** | TRINITY_DN16676_c0_g2 | 0 | 0 | 0 | 3.9 | 2.93 | 2.89 | 8.31E-05 | Predicted Protein |
| **98** | TRINITY_DN1794_c0_g1 | 0 | 0 | 0 | 3.92 | 2.16 | 3.82 | 0.000175 | Predicted Protein |
| **99** | TRINITY_DN180254_c0_g1 | 0.01 | 0 | 0 | 4.85 | 4.11 | 5.09 | 7.03E-05 | Predicted Protein |
| **100** | TRINITY_DN30545_c1_g1 | 0.08 | 0.08 | 0.06 | 16.07 | 11.96 | 18.36 | 0.000123 | Predicted Protein |

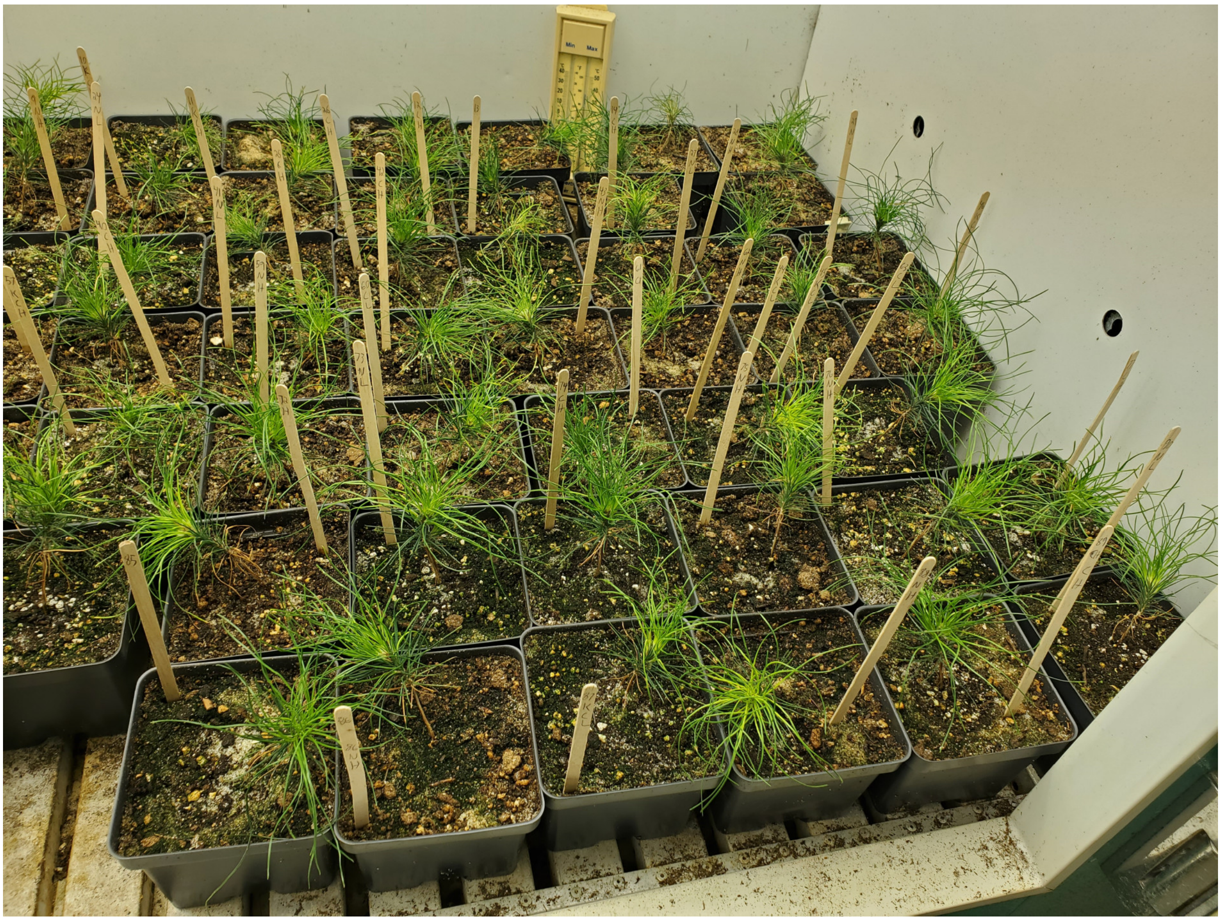

**Figure S1**. *Pinus banksiana* seedlings water growing in a growth chamber after treatments with nickel sulfate, potassium sulfate, and water.
